# MOSAIC - A Unified Trait Database to Complement Structured Population Models

**DOI:** 10.1038/s41597-023-02070-w

**Published:** 2023-06-01

**Authors:** Connor Bernard, Gabriel Silva Santos, Jacques A. Deere, Roberto Rodriguez-Caro, Pol Capdevila, Erik Kusch, Samuel J. L. Gascoigne, John Jackson, Roberto Salguero-Gómez

**Affiliations:** 1 Department of Biology, University of Oxford, 11a Mansfield Rd, OX13SZ Oxford, United Kingdom; 2 Department of Ecology, Rio de Janeiro State University, 20550-900 Rio de Janeiro, Brazil; 3 National Institute of the Atlantic Forest (INMA), 29650-000 Santa Teresa, Espírito Santo Brazil; 4 Department of Evolutionary and Population Biology, Institute for Biodiversity and Ecosystem Dynamics, University of Amsterdam, 1012 WX Amsterdam, Netherlands; 5 Departamento de Biología Aplicada, Universidad Miguel Hernández. Av. Universidad, s/n, 03202 Elche (Alicante), Spain; 6 School of Biological Sciences, University of Bristol, 24 Tyndall Ave, Bristol, BS8 1TQ United Kingdom; 7 Center for Biodiversity Dynamics in a Changing World (BIOCHANGE), Arhus University, Aarhus, Denmark; 8 Section for Ecoinformatics & Biodiversity, Department of Biology, Arhus University, Aarhus, Denmark; 9 Centre for Biodiversity and Conservation Science, University of Queensland, St. Lucia, QLD Australia; 10 Evolutionary Demography Laboratory, Max Plank Institute for Demographic Research, Rostock, Germany

**Keywords:** Population dynamics, Data integration, Evolutionary ecology, Ecophysiology, Biogeography

## Abstract

Despite exponential growth in ecological data availability, broader interoperability amongst datasets is needed to unlock the potential of open access. Our understanding of the interface of demography and functional traits is well-positioned to benefit from such interoperability. Here, we introduce MOSAIC, an open-access trait database that unlocks the demographic potential stored in the COMADRE, COMPADRE, and PADRINO open-access databases. MOSAIC data were digitised and curated through a combination of existing datasets and new trait records sourced from primary literature. In its first release, MOSAIC (v. 1.0.0) includes 14 trait fields for 300 animal and plant species: biomass, height, growth determination, regeneration, sexual dimorphism, mating system, hermaphrodism, sequential hermaphrodism, dispersal capacity, type of dispersal, mode of dispersal, dispersal classes, volancy, and aquatic habitat dependency. MOSAIC includes species-level phylogenies for 1,359 species and population-specific climate data. We identify how database integration can improve our understanding of traits well-quantified in existing repositories and those that are poorly quantified (*e.g*., growth determination, modularity). MOSAIC highlights emerging challenges associated with standardising databases and demographic measures.

## Background & Summary

The ecological sciences have recently joined the open data revolution^1–3^. As a result of initiatives promoting open data, total species distribution records measure in the hundreds of millions^4,5^. Functional trait data exist for tens of thousands of species across the globe^6–8^. Global distributed networks, remote sensing, and other ecological sensor data networks are feeding information into the open data space, and we are experiencing a rapid increase in the number of ecological databases. The growth of open data is reflected in state-of-the-art climate models (*e.g*., ERA-5 [https://www.ecmwf.int/en/forecasts/dataset/ecmwf-reanalysis-v5]) becoming available at fine spatial and temporal resolutions suitable for biological analyses^9,10^, the growth of behavioural trait datasets^11^, and large population datasets (Living Planet Data, successor to the Global Population Dynamics Database [http://livingplanetindex.org/data_portal]; Human Mortality Database;^12^ Human Fertility Database;^13^ AnAge Database;^14^ DATLife [Max Plank Institute of Demographic Research 2022; https://datlife.org/]). Data access and scaling has extended to biological complexity at the ecological community ecology level (BioTime;^15^ Web of Life;^16^ metaCommunity Ecology: Species, Traits, Environment and Space (CESTES^17^); Environmental Data Initiative [https://environmentaldatainitiative.org/]). The growth of records in ecology datasets and complementary environmental datasets is enabling us to test ecological theory at larger and more complex scales. In line with the expansion of open access data practices, however, there is a need to improve and coordinate data standards to guide the systematic collection and management of data across different trait collection programmes^18–20^.

Despite the increased availability of biological data, synthesizing datasets for analysis is hampered by the lack of complementarity between databases. The rise of data sharing and proliferation of databases can encourage a fragmented and decentralized information landscape unless there is active coordination. Interoperable data systems reflect continuity in the format of data types and structure to allow compatibility across computers and software. Converting datasets into interoperable formats may require the transformation of constituent data types into standardized spatial, temporal, and measurement scales, accounting for known differences/biases in methods^21^. The need to improve interoperability across datasets is demonstrated by the widescale emergence of data harmonization initiatives across fields of ecology^22–24^. In the past decade, dozens of initiatives have taken shape to both centralize data from existing datasets and to improve data interoperability: standardising units, scales, and terminology for comparative purposes^25,26^. Unifying data formats and streamlining their integration unlocks the potential for existing datasets to answer questions that cut across levels of biological complexity. Linking together levels of biological complexity is critical for identifying how phenomena emerge and transmit across different levels of biological organisation, upscaling and downscaling through biological systems. For example, the critical linkages between genetics and biochemistry^27^, biochemistry and physiology^28^, and physiology and demography^29,30^ have benefitted from dataset integration.

The limitations of global-scale datasets are shifting away from data availability and toward data interoperability. For functional traits, momentum toward data integration is evidenced by recent database initiatives^31^ and global networks that, like the Open Trait Network (https://opentraits.org/), aim to standardize and integrate trait data across taxa^18^. Despite major improvements in the consolidation and accessibility of trait data, there is not yet a single-source centralized database spanning behaviour, physiology, habitat, and other trait data for a wide range of species. Existing databases are often linked by taxonomy (*e.g*., FishBase^32^, CoralTraits^33^, MammalBase^34^, AmphiBio^35^); trait type (*e.g*., Tree of Sex^36^, TreeBase^37^, Xylum Functional Traits^38^); data type (*e.g*., GBIF (https://www.gbif.org/), MOL^39^, TetraDensity^40^); or a combination of the taxonomies and traits (WooDiv^41^, CarniDiet^42^). A number of other databases take a more general approach in their thematic scope, but are still constrained to a limited set of traits and taxonomy (*e.g*., Amniote^43^, Pantheria^44^, BIEN^7,45^, TRY^23^). MOSAIC offers a platform that integrates databases in the remit of species with structured population models across their ecological traits.

Here, we introduce MOSAIC, a centralized database of trait data across the Tree of Life. MOSAIC is an open-access database that complements the existing demographic data available in the COMPADRE Plant Matrix Database^46^, COMADRE Animal Matrix Database^47^, and the new PADRINO IPM Database^48^. MOSAIC v. 1.0.0 includes 14 frequently used traits that encompass morphological, reproductive, dispersal, and habitat type attributes for some 300 species of animals and plants. Additional traits will be added in the future (see *Future Direction, below*). MOSAIC allows users to integrate structured population data to probe larger questions through the collection, curation, and complementarity of relevant contextual data.

## Methods

### Scope and coverage of MOSAIC

**Fig. 1:**
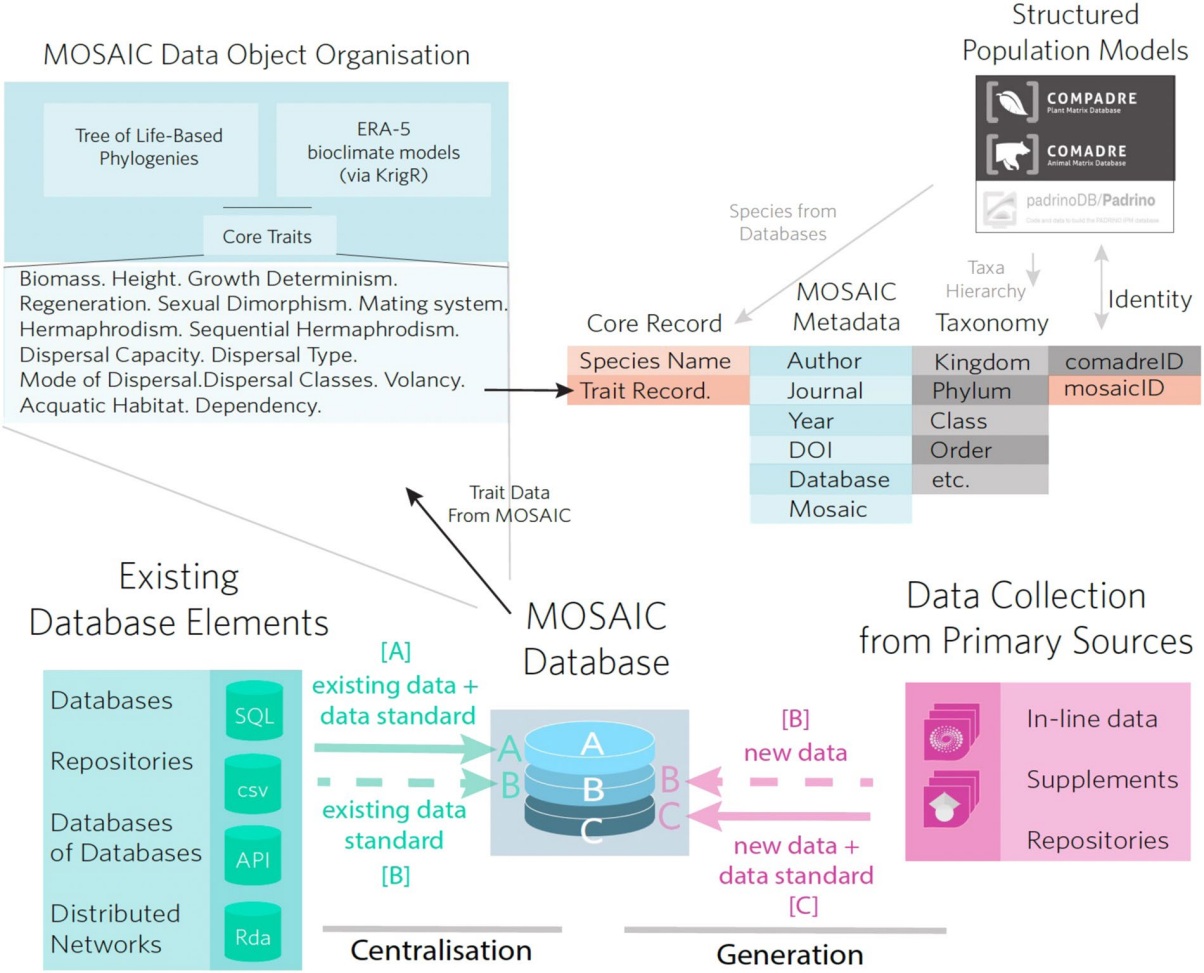
Structure of the MOSAIC database (v1.0.0). The MOSAIC database comprises a combination of existing trait records centralised from data servers, databases, and datasets and new records collected from the primary literature by the MOSAIC team. Existing records are labelled, MOSAIC-A, in the mosaic metadata attribute field and include provenance in the metadata. New data records that adhere to existing data standards and that fit into the remit of an existing database (*i.e*., a data gap filled by the MOSAIC team) are labelled MOSAIC-B. New records that do not have an existing data standard or for which there is not an existing database are labelled MOSAIC-C.

**Fig. 2:**
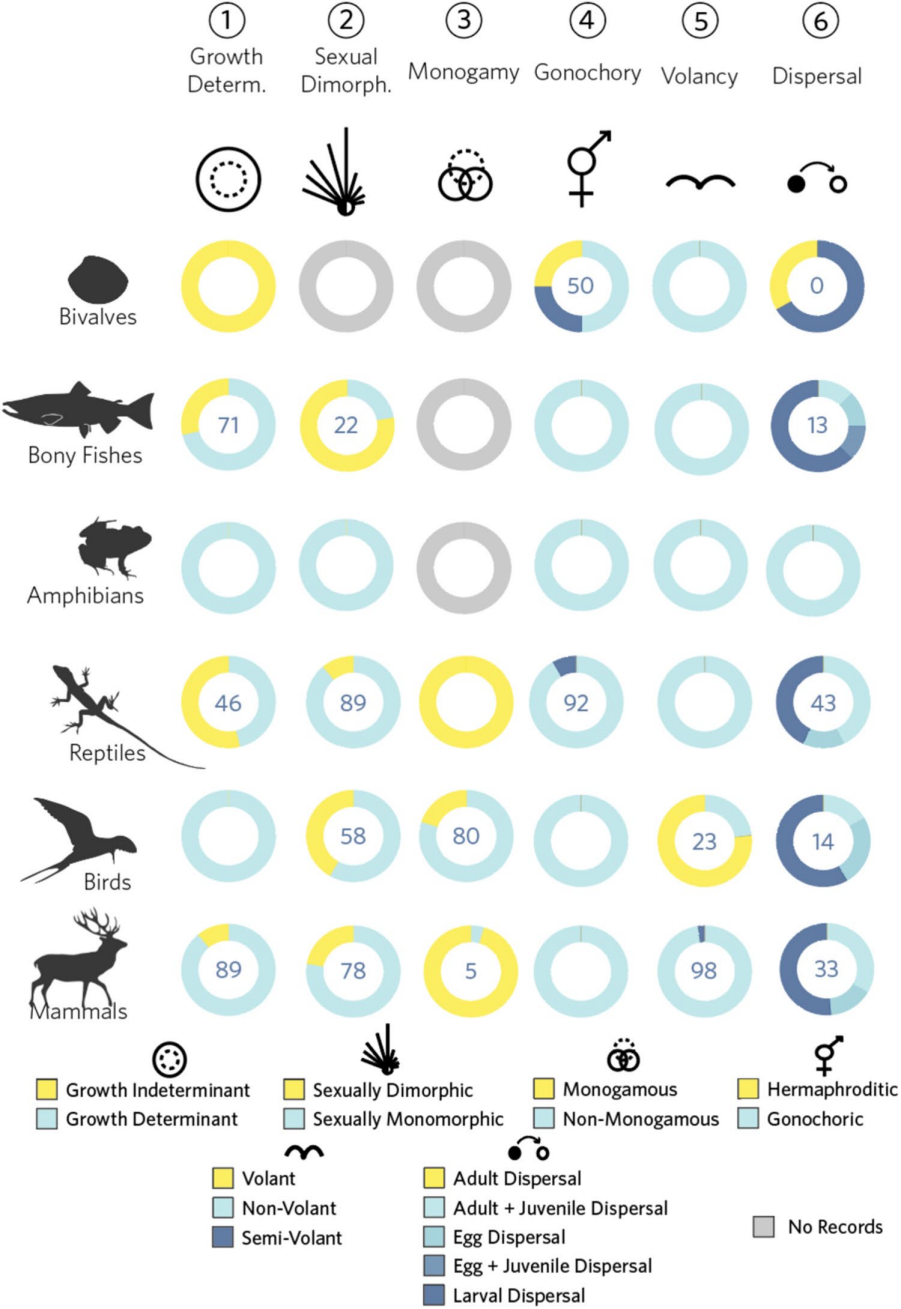
Trait fields in the MOSAIC database (v1.0.0). The MOSAIC database contains a combination of continuous and discrete trait fields. Six of the 14 trait fields are illustrated here for animals, and organised by trait level (all discrete fields) and taxonomic classification. Trait distributions vary strongly by trait and taxonomy. Observed variation in trait values by taxonomic group is a prerequisite condition to their potential value toward explaining reported variation in vital rates and resulting demographic properties across the Tree of Life. Species included in the MOSAIC database are limited to those for which stage-structured population data exist in the COMADRE, COMPADRE, and PADRINO databases.

**Fig. 3:**
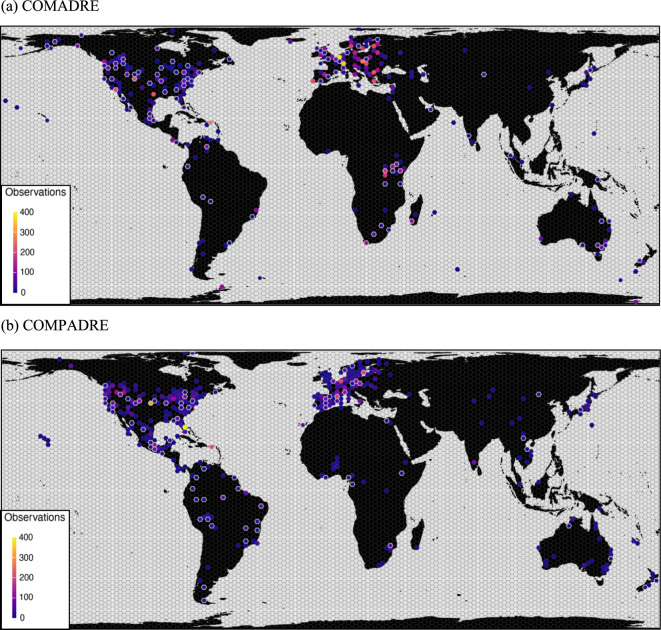
The spatial distribution of MOSAIC (v1.0.0) records relative to the (a) COMADRE and (b) COMPADRE databases. The COMADRE and COMPADRE dataset include animal and plant demographic data, respectively, on all continents except Antarctica, with a substantial bias to North America and Central Europe. MOSAIC introduces trait records that include models in all major geographies of COMADRE and COMPADRE. The map shows the density of matrix population models globally (color graded by density per area in *ca*. 150 km^2^ hexagonal cells). Cells bordered by white represent localities of population models for which there are trait records included in the first release of the MOSAIC trait database. Species prioritised in v1.0.0 of MOSAIC did not include the PADRINO dataset, so no distribution figure is shown for integral projection models.

**Fig. 4:**
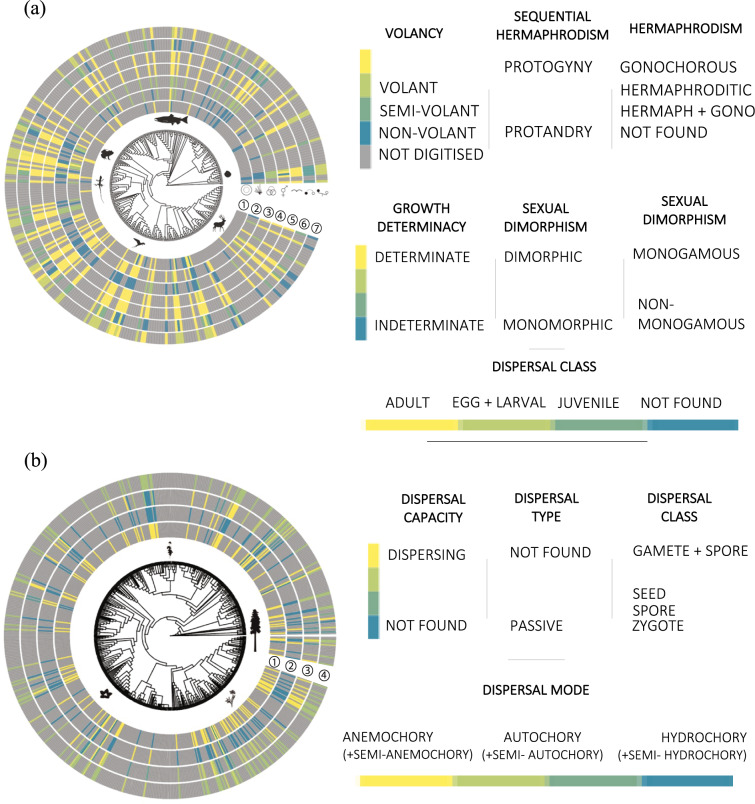
Phylogenetic mapping of traits included in MOSAIC (v1.0.0). (**a**) Phylogenetic distribution of seven major traits for animals: (1) growth determination; (2) sexual dimorphism; (3) monogamy; (4) gonochory; (5) volancy; (6) dispersal; and (7) biomass. (**b**) Phylogenetic distribution of four traits in plants: (1) dispersal capability; (2) dispersal type; (3) dispersal mode; and (4) dispersal class. Figures developed in R using the ggtree package.

**Fig. 5:**
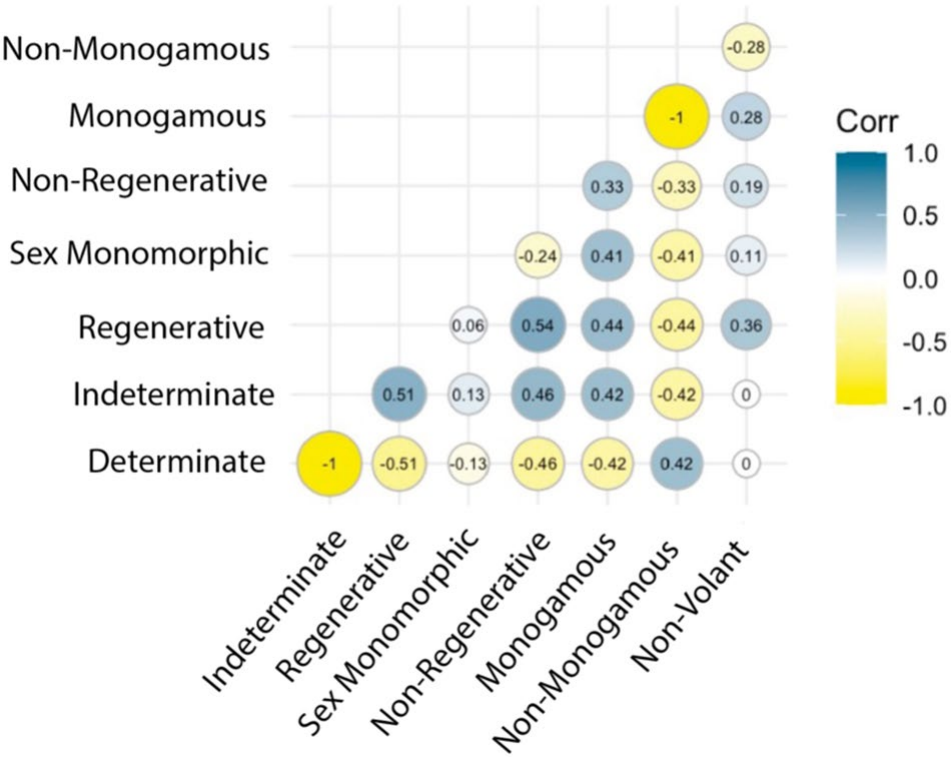
Trait covariance in the MOSAIC database (v1.0.0). Some traits exhibit high correlation in the MOSAIC database, such as between growth determination and between growth regeneration. Trait associations are expected to occur in the MOSAIC dataset and may reflect widespread constraints or statistical anomalies, particularly when dealing with small samples or specific taxonomic subgroups. Trait covariation can be symptomatic of biomechanical constraints (*e.g*., flight and biomass), major growth characteristics (*e.g*., modularity and growth determination), or other past or presently compelled associations (*e.g*., height and vessel density).

The MOSAIC database (v. 1.0.0) includes 14 key trait records across 300 species (Fig. 1). MOSAIC is designed to provide complementary data for analysis in connection with structured population models: matrix population models (MPMs^49^), where state variables are discrete (*e.g*., age^50^, ontogeny/development^51^, discrete classes of size^52^), and integral projection models (IPMs^53^), where the state variables are continuous (*e.g*., size^54^, mass^55^, parasite load^56^). The traits included in MOSAIC 1.0.0 were identified as a set of physical, physiological, geographic, and behavioural attributes of most immediate relevance to demographic research (see Table 1; more information at https://mosaicdatabase.web.ox.ac.uk). Traits were also selected in consideration of the lack of standardized and centralized databases for certain traits (*e.g*., volancy, modularity, and growth indeterminacy; see Fig. 2 for trait variance and taxonomic structure of select traits excluded from existing databases). Importantly, we note that MOSAIC is not a general dataset for analysis of functional traits, as this is available through other extensive repositories (*e.g*., TRY^23^, BIEN^7,45^). Instead, the focus of MOSAIC is on providing taxonomic coverage to species with open-access structured population models available in the COMADRE^47^, COMPADRE^46^, and PADRINO^48^ databases (See Figs. 3, 4 for spatial scope and taxonomic scope with respect to structured population databases, respectively). MOSAIC provides a much-needed interoperability between existing databases that are relevant to demography. By doing so, MOSAIC helps to fill critical data needs of population ecologists and functional trait ecologists (see Fig. 5 for relevant covariance structure).

**Table 1:** Variable names and meaning contained in the MOSAIC Database, organised into seven general trait domains.

Aspect	Variable	Description	Units
Taxonomy	1 *Species Author*	Taxonomic species name as used by the author(s) in the publication. When more than one study exists for the same species, these are given sequential numeric suffixes (e.g. Ursus_americanus, Ursus_americanus_2, etc.)	NA
(Parallels COM(P)ADRE)	2 *Species Accepted*	Currently accepted taxonomic name according to the Catalogue of Life (www.catalogueoflife.org). See the Supplementary Online Material S3 for an R script to check accepted and synonym names from SpeciesAuthor above	NA
	3 *Common Name*	English common name of SpeciesAccepted	NA
	4 *Family*	Taxonomic family of study species	NA
	5 *Order*	Taxonomic order of study species	NA
	6 *Class*	Taxonomic class of study species	NA
	7 *Phylum*	Taxonomic phylum of study species	NA
	8 *Kingdom*	Taxonomic Kingdom of study species	NA
Species information General	9 *Organism type*	General plant/algae type, based mainly on architectural organisation. The species was assigned to one of these possible values using the description of plant growth type provided by the author and other external sources (e.g. other publications)	Categorical/ Factorial
	10 *DicotMonocot*	Whether species is a dicot or monocot	Categorical/Factorial
	11 *AngioGymno*	Whether species is an angiosperm or a gymnosperm	Categorical/Factorial
Source information	12 *Authors*	Surname (family name) of all author	NA
	13 *Journal*	The document from which data were sourced.	NA
	14 *YearPublication*	Year of publication	NA
	15 *DOI*	Digital Object Identifier number	NA
Traits	01 *Biomass*	Mean mass of an individual/whole-organism. (Plants) Plant mass is measured as aboveground dry mass. See “Further information” for additional information on belowground biomass.	Grams
	02 Height	(Plants) Mean height of the whole organism/whole individual from surface (i.e. substrate) to tallest vertical extremity. (Animals) Mean ventro-dorsal length. (i.e. for terrestrial quadrupeds, vertical distance from the ground to the top of the shoulder. For marine bony fishes, from the top of the high point of the dorsal fin to the base of the ventral ridge or ventral fin, whichever is a longer distance perpendicular to the anterior-posterior axis.)	Centimetres
	03 Growth Determination	Whether a species exhibits growth determinacy or not.	Categorical/Factorial
	04 Regeneration	Capacity for an individual to regenerate any substantial part of its body, including autotomy. Autotomy is defined as “The voluntary severance by an animal of a part of its body (commonly one of its own limbs), usually to escape capture by a predator that has seized that part. The part then regrows.”	Categorical/Factorial
	05 Sexual Dimorphism	An indicator of whether sexual dimorphism is exhibited in the species. Sexual dimorphism is defined as “the occurrence of morphological differences (other than primary sexual characters) that distinguish males from females of a species of organism.” (Oxford Dictionaries of Ecology and Zoology)	Categorical/Factorial
	06 Mating System	System of mating; the organisation of sexual interactions of individuals within populations based on sex.	Categorical/Factorial
	07 Hermaphrodism	Indicator of whether a species exhibits hermaphrodism or monoeicieosity. Hermaphrodism is defined as: “An individual that possesses both male and female sex organs; i.e. it is bisexual.” (Oxford Dictionary of Zoology). Monoeciousness is defined as: “Applied to an organism in which separate male and female organs occur on the same individual (e.g. to a plant which bears male and female reproductive structures in the same flower or separate male and female flowers on the same plant, or to a hermaphrodite animal). Some authors restrict the term botanically to plants with separate male and female flowers; plants which bear male and female reproductive organs in the same flower are then called hermaphrodite.”	Categorical/Factorial
	08 Sequential Hermaphrodism	Indicator of whether a species is protogynous or protandrous.	Categorical/Factorial
	09 Dispersal Capacity	An indicator for whether or not a species exhibits dispersal behaviour or at any stage in its life cycle. Where dispersing, a categorical description of whether dispersal is natal or breeding or otherwise. Dispersal is defined as “The tendency of an organism to move away, either from its birth site (natal dispersal) or breeding site (breeding dispersal): the opposite of philopatry.” (Oxford Dictionary of Zoology).	Categorical/Factorial
	10 Type of Dispersal	An indication of whether dispersal is a passive (requires assistance) or active (no assistance) event. See DispClasses for more information	Categorical/Factorial
	11 Mode of Dispersal	An indicator of the mode of dispersal of the species (plant and animal specific terminology). (*e.g*., phoretic dispersal in *Daphnia magna*).	Categorical/Factorial
	12 Dispersal Classes	Age- or stage-classes of the species that are capable of dispersal.	Categorical/Factorial
	13 Volancy	An indicator of whether a species is volant or non-volant (i.e., able to fly or not).	Categorical/Factorial
	14 Aquatic Habitat Dependency	An indicator of whether a species is dependent on water or not. (e.g., limnic, lentic, lotic)	Categorical/Factorial

A more detailed description can be found in the MOSAIC user guide found at: https://mosaicdatabase.web.ox.ac.uk/.

### Data sources

MOSAIC is both a *meta-*database (a database of databases) and a database in its own right, containing new trait records from primary literature (Fig. 1). The MOSAIC database contains records centralized from existing datasets where functional traits relevant to population ecologists can be openly accessed and redistributed (*e.g*., BIEN^7,45^). Licensing terms of these databases are included in the supplement (Appendix S1: Database Licensing Terms for Constituent Databases of MOSAIC). The records reflected in the MOSAIC database do not encompass the entirety of the source databases but are instead partial facsimiles of those databases that reflect records relevant to demographic databases (COMADRE, COMPADRE, PADRINO). MOSAIC has three major components: (1) trait records sourced from existing databases (22%); (2) trait records newly procured through searching the primary literature (71%); and (3) trait record markers indicating the presence of records in non-open-access database (7%). MOSAIC trait markers exist for one of two reasons: the database containing the records of interest does not allow records to be accessed or limits redistribution rights behind individual registration and/or specific use applications; or records in other databases contain multiple records for a species, which do not currently fit within the data structure of version 1.0.0 of MOSAIC (see *Future Targets for MOSAIC*).

In addition to identifying whether a trait record is new, the “MOSAIC” attribute field also identifies whether the attribute field (*i.e*., trait name) is part of an existing database. For example, the MOSAIC field might indicate that a record for specific leaf area is new for a specific species, and also that the attribute is part of databases such as TRY^23^ or BIEN^7,45^. By contrast, a new species record for volancy would indicate that there are currently no databases that systematically collect data on volancy attributes and therefore that all volancy records in the MOSAIC database are new.

### Organisation of sources in MOSAIC

The MOSAIC attribute field is a factorial variable with three levels: MOSAIC-A, MOSAIC-B, and MOSAIC-C. The first of these levels, MOSAIC-A (Fig. 1), labels only records that reflect existing databases (*i.e*., provenance of an existing data acquisition service); the second, MOSAIC-B, labels new records collected by the MOSAIC team that are in a trait field within the scope of an existing database initiative (*e.g*., specific leaf area in BIEN); and the third, MOSAIC-C, labels new records collected by the MOSAIC team on traits for which there is not currently a database initiative centralising records. If a datum has been adopted from another dataset or database, then the relevant source is referenced in the *Database* attribute column. Note that this value will be “NA” for all MOSAIC-C records, logically. Over time, data sharing will move records in MOSAIC-B to MOSAIC-A as the MOSAIC-B traits are assimilated in the database networks that specialize on an existing trait (*i.e*., data feedback; see Fig. 1).

### Sources and provenance of records

Because of existing limitations on data access, some datasets cannot be transferred into MOSAIC. Where data exist outside of the MOSAIC platform, but have restricted access, the MOSAIC database directs users toward the appropriate database on a trait and taxa-specific level (see metaMOSAIC). The *MOSAIC User Guide* (Appendix S2) explains differences between data gaps that are yet to be reviewed and those that are true gaps (*e.g*., volancy/flight capability of plants). Data were obtained through searching peer-reviewed records and PhD dissertations of ISI Web of Science, Scopus, and Google Scholar using key words pertinent to the species name and trait field in question (see Appendix S3 for a list of keywords queried). The archives of data repositories, including the Figshare digital repository^57^ (https://figshare.com/articles/dataset/MOSAIC_trait_database/21035857; see Appendix S3 for a complete list of repositories reviewed – to be maintained hereafter on the MOSAIC portal) and journal archives that have a high occurrence of data publishing, including *Nature Scientific Data*, *Methods in Ecology and Evolution*, and *Journal of Ecology* were carefully reviewed. MOSAIC also reflects a review of data from data aggregating servers, such as the open traits network (https://opentraits.org/), the ecological data wiki (https://ecologicaldata.org/), environmental data initiative (https://environmentaldatainitiative.org/), and databases that aggregate other databases (*e.g*., BIEN^7,45^ and TRY^23^). The complete set of key words used in this review is detailed in a supplement to the *User Guide* (Appendix S3). A current list of databases reviewed in the development of MOSAIC is included in Appendix S4. Suggested data sources and key terms can be submitted through the MOSAIC data portal.

### metaMOSAIC: licensed data, access limitations, and restricted redistribution of existing records and databases

Not all datasets permit open use, dissemination, and redistribution of their trait data. Where limitations on the data collation and redistribution apply, there may be application procedures, registration, and other actions necessary for an individual to obtain access to specific trait records for analysis (*e.g., TRY*). MOSAIC centralizes the metadata for datasets that do not allow data to be redistributed to help navigate to relevant data resources outside the scope of open access. Records in limited access databases can be searched in MOSAIC by taxonomic group and by trait. MOSAIC links researchers to application materials for requesting access to those limited-access databases. The data access of licensed or non-open-access databases is stored in a data object called metaMOSAIC that is an extension of the MOSAIC database. Thus, the MOSAIC dataset provides data where it is accessible and metaMOSAIC guides researchers to where data exist with registration. When searching fields in the MOSAIC database, the metaMOSAIC adjunct dataset indicates if data are available in these ancillary sources (see *User Guide* in Appendix S2 for more information) and provide links to pertinent sources and data through the provider. metaMOSAIC is part of the main database object accessible through the MOSAIC portal.

### Database updates

The MOSAIC database will be updated as new data are added to the COMADRE, COMPADRE, and PADRINO databases. Updates of the MOSAIC database will account for newly discovered data sources and new literature that adds to or changes the species-level traits in the database, as well as correct errors from earlier versions. New MOSAIC versions will be released periodically with a notice published on the website, in the data object metadata, the mosaic GitHub page (https://github.com/mosaicdatabase), and through updates in associated packages in conformance with standard semantic versioning (a three-part version code reflecting major, minor, and patch updates, in respective positions). Updates will be published to the mosaic portal (https://mosaicdatabase.web.ox.ac.uk), social media (Twitter: @mosaicdatabase), and GitHub (https://github.com/mosaicdatabase). The MOSAIC portal includes a location for submission of recommendations for additions or suggested changes to the database.

### Future targets of MOSAIC

#### Versioning

MOSAIC database updates and version history will be posted to the MOSAIC portal in the future.

### Taxonomic scope

Future versions of MOSAIC are scheduled for expansion to include 50 core traits across some 1,500 species (the current entirety of COMPADRE, COMADRE, and PADRINO; *MOSAIC target traits*; see *User Guide* [Appendix S2]). The MOSAIC core traits list will guide the future roll-out of attributes gathered and stewarded by the MOSAIC team, but additional trait suggestions can be submitted through the online portal.

### Interspecific variation

Future versions of the MOSAIC database will adopt a file structure that will accommodate multiple records per species. Once records for COMADRE, COMPADRE, and PADRINO are fully populated across the MOSAIC traits with mean, pooled, or other representative quantities (*e.g*., mean leaf size for all plants or adult bodymass of animals), secondary records will be added. Existing trait databases may contain multiple records per species (see, for example, structure of COMADRE^47^, COMPADRE^46^, BIEN^7,45^, TRY^23^), although some databases host a single record per species’ trait, such as age and growth rate for animals in AnAge^14^. To facilitate research into intra-specific trait variation^58–60^, MOSAIC provides provenance of records, whether records were subject to selection or merger (means or pooling), and fields that identify whether multiple records are known to exist. Where records for a given species were isolated from existing databases, mean values are often retrieved, and the database sourcing additional data is noted in the database under the attributed field (“Additional Trait Data Available” field).

### citMOSAIC: Citizen science

In addition to metaMosaic, which guides users to licenced data not reported in the MOSAIC database, MOSAIC plans to roll out a database of identical structure to MOSAIC that gathers information from citizen science datasets. Like MOSAIC, citMOSAIC will have three components (citMOSAIC-A, citMOSAIC-B, citMOSAIC-C), reflecting the same relationship of databases and fields to MOSAIC. citMOSAIC will be kept independent of the main MOSAIC database to avoid conflation of peer reviewed literature and PhD dissertations from citizen science data. Where appropriate to use datasets together, the metadata, query functionality, and design of citMOSAIC will mirror that of MOSAIC to promote interoperation of databases. citMOSAIC will be downloadable from the MOSAIC portal website.

Data Records

The current version of MOSAIC (v. 1.0.0) is deposited as series of flat data files (comma separated values and text files) in Figshare (https://figshare.com/articles/dataset/MOSAIC_trait_database/21035857). The data in this paper and complete database are also published on the MOSAIC website https://mosaicdatabase.web.ox.ac.uk and associated GitHub repository https://github.com/mosaicdatabase/mosaicdatabase. All data is open access without restrictions on access.

The MOSAIC database can be downloaded directly into R as a series of flat tabular data (comma separated values and text files) on Figshare (https://figshare.com/articles/dataset/MOSAIC_trait_database/21035857) and, optionally, as an S4 object (see *Usage Notes—Accessing MOSAIC*). Future versions of the MOSAIC datasets with any corrections and new data records will be updated to the GitHub page (github.com/mosaicdatabase/mosaicdatabase) linked with the direct download in R and updated to Figshare. Data use and redistribution is covered by a Creative Commons CC-BY license, with unrestricted use and modification with attribution, consistent with MOSAIC’s constituent databases (Appendix S1).

Description of the individual trait fields are included in Table 1. Detailed discussion of each data field, units, precision, and cautionary notes included in the MOSAIC *User Guide* (Appendix S2). In its first version (v. 1.0.0), MOSAIC data has 41% density coverage across 14 core trait fields of approximately 300 species. MOSAIC provides 100% density of climate data for all species in COMADRE, COMPADRE, and PADRINO for which there are GPS locations (86% of all records across the databases). 71.9% of species in COMADRE, COMPADRE, and PADRINO had records in the Online Tree of Life phylogeny included in MOSAIC. Data completeness varies greatly amongst trait fields, as does the sourcing of data from primary and secondary datasets (Fig. 2).

Across formats, each tabular row or vector element represents a single species. Columns are representative of attributes or metadata associated with attributes. Unique identifiers associated with the matrix population models and integral population models in COM(P)ADRE and PADRINO databases, respectively, to link demographic models with trait records. In the current MOSAIC version (v. 1.0.0), only one record per species is released, but future versions will incorporate multiple records per species.

## Technical Validation

The technical validity of records in the MOSAIC database is based on three levels of review. First, all records in MOSAIC, regardless of whether they are collected from primary or secondary data sources, are obtained from either peer reviewed journals or scholarly equivalent documents (*e.g*., PhD dissertation). Second, all data sources in MOSAIC were reviewed to ensure that they are reasonably representative of the trait record for the species (e.g., screened for sample size, geography relative to the pertinent population record(s), and methods suitable to the trait). Third, MOSAIC will be periodically reviewed, added to, and amended, additive to any revisions that might come about through the feedback portal. As a result, MOSAIC will be adaptively managed to ensure that all records are of suitably high-quality and grounded in peer-reviewed data. MOSAIC also contains metadata for all records, ensuring provenance to original records and secondary standardisation.

### Peer-reviewed data

All data in MOSAIC are either published in a peer reviewed academic journal, PhD dissertation, or other peer reviewed source (e.g., Oxford Bibliographies). Unique identifiers for the publications that source the data is provided for every record. Prior to the review of the record by the MOSAIC team, all trait records were technically scrutinized and reviewed by more than one subject area expert. In addition to peer review of the publication record, many MOSAIC records are sourced from existing databases that impose technical standards beyond those imposed by the initial publication and have technically assessed the quality of underlying data.

### Record review and selection

The MOSAIC team evaluated whether records were consistent across the literature. Where multiple records were identified containing different numeric values for a continuous trait, the MOSAIC team selected a focal record based on: (1) sample size, (2) geographic extent, (3) geographic proximity to the site, and (4) method of measurement. If multiple records were identified for factorial traits that were in conflict with one another, then those records were excluded from the initial release of MOSAIC v.1.0.0. Only unanimously consistent factorial trait values were included in the first version of MOSAIC. At least two members of the MOSAIC team assessed records in every case to ensure that there was agreement in the sourcing of the data.

### Quality checks

The MOSAIC team reviewed the data for any outliers. In addition to reviewing for general anomalies from the data variance structure, as part of the data collection protocol, the MOSAIC team screened data for bounds applicable to each trait (see *User Guide*, Appendix S2). These value bounds ensured that all reported data were within the range of biologically realistic values as an additional safeguard against spurious values. Beyond confirming the general data structural integrity, the MOSAIC team compared the overlay of records in COMADRE, COMPADRE, and PADRINO with the source data for MOSAIC to ensure congruence of the data transferred from source databases. All automated data overlays were manually checked for their integrity against source data.

### Ongoing development & growth

The MOSAIC database will continue to grow and, in the future, will accommodate multiple trait records per species. This trait record redundancy will provide another level of protection against spurious records, in addition to providing quantification of trait variance. Future database development plans include a schedule to incorporate GPS coordinates to formalize the distance between each trait record and the corresponding population records.

### Metadata

The MOSAIC database contains the source data for each trait record, including (1) Author(s), (2) Year of Publication, (3) Journal of Publication, (4) DOI/ISBN of the publication, and (5) Inclusion in other databases. Future versions of the database may contain additional metadata for the trait data.

## Usage Notes

### Accessing MOSAIC

A guide for downloading the MOSAIC database can be accessed in the Supplementary Material (Appendix S2). The MOSAIC database is open access and can be downloaded into R as a csv using the below line of code:

read.csv(“ https://raw.githubusercontent.com/mosaicdatabase/mosaicdatabase/main/MOSAIC_v1.0.0.csv ”)

The MOSAIC database is a comma separated value (csv) format that is publicly archived under a CC-BY-4.0 license accessible from Figshare (https://figshare.com/articles/dataset/MOSAIC_trait_database/21035857), GitHub (https://github.com/mosaicdatabase/mosaicdatabase), and the MOSAIC portal (https://mosaicdatabase.web.ox.ac.uk). Phylogenetic data is formatted for use and manipulation using the open-source R-package ape (Analyses of Phylogenetics and Evolution; Schliep & Paradis 2019) which can be installed by CRAN.

Mosaic *User Guide*, *Vignette*, and *Data Collection Protocols* are all included in the Supplemental materials (Appendix S2, S3, and S5). The *User Guide* specifies the classifications, precision, and data types for each trait field. The MOSAIC *User Guide* details the metadata on the structure of data for each trait (*e.g*., species, genus, mosaic index). An updated list of the databases that are directly or indirectly addressed by the MOSAIC database is maintained on the MOSAIC portal.

### Field values

MOSAIC records contain one of three values: “NA,” “NDY,” or a trait value that can be numeric, factorial, or a character string, as discussed further in the User Guide. NAs apply to fields that have been reviewed and which do not apply to the species of interest. For example, plants do not have flight capacity, and thus NAs apply to them for this trait. Likewise, height and canopy size are key morphological dimension of plants that may not transfer meaningfully to most vertebrates, where adult biomass is a more relevant^61,62^ and oftentimes used trait. By contrast, NDYs represent “Not Digitized Yet”, indicating fields/species that have not been reviewed for potential records. All other values will reflect the units described in the User Guide.

### Database navigation

The *User Guide* contains detailed information on navigating the MOSAIC R data object. The primary MOSAIC object contains species-specific attribute values for 14 traits. Climate data and phylogeny are independent files accessible in the same locations described above (csv and phylo formatted, respective). Climate is an independent file because it is based solely on model-specific coordinates (and therefore has multiple values per species). Phylogeny is an independent file because it is a phylo object (a special kind of list object in R accessed through ape) and therefore is not in a format amenable to species-specific csv summary.

The primary MOSAIC file contains 14 fields and can be queried through S3 syntax. Rows represent species and are included in the column titled: species_accepted. Columns represent attributes that are either the trait value or trait metadata. The base format for accessing data is: mosaic$trait.name. In the aforementioned syntax, the trait.name should reflect one trait (*e.g*., volancy). Metadata can be accessed with the syntax: mosaic$trait.name_metadata, where metadata is replaced by the name of the metadata field (e.g., journal, doi, author). Species names are specified in conformance with the Catalogue of Life (www.catalogueoflife.org), also consistent with the COMPADRE, COMADRE, and PADRINO databases. The *User Guide* provides specific guidelines for querying fields within the data object in R and for negotiating the dataset in finer detail (Appendix S2).

### Error reporting

Users can submit errors for correction by email to: mosaicdatabase@biology.ox.ac.uk. The MOSAIC portal also has an *Error Report* page for reporting potentially erroneous records, or to query additional questions (https://mosaicdatabase.web.ox.ac.uk/suggested-additions-error-reporting; but see also FAQ: https://mosaicdatabase.web.ox.ac.uk/frequently-asked-questions). Potential errors can be reported anonymously or with contact information (*e.g*., name, email). Decisions on reported errors will be disclosed on the Error Report page (https://mosaicdatabase.web.ox.ac.uk/suggested-additions-error-reporting) and to the reporting party if contact information is included in the request.

### Recommended records

Users can submit records by email to: mosaicdatabase@biology.ox.ac.uk. The MOSAIC portal also has a *Recommended Records* page (https://mosaicdatabase.web.ox.ac.uk/suggestion-additions) for reporting suggested records that are not included in the MOSAIC database. Recommendations can be made with or without contact information. Contact information will be used exclusively for clarifying questions and updating the commenter when records are included. MOSAIC will report decisions on the *Recommendations Incorporated* page (https://mosaicdatabase.web.ox.ac.uk/suggestion-additions). Users may also request new data fields to be prioritized in future rollouts. Given the realities of limiting resources, The MOSAIC team will do their best to include the requested records in future versions.

### Cautionary notes

Records in the MOSAIC database are gathered and standardized under the protocols detailed in the *User Guide* (Appendix S2; also available through the MOSAIC portal). Users should be attentive to the precision, levels, and context of data in MOSAIC when used for analysis. For those records in the dataset that come from multiple individuals, we present them as statistical components (*e.g*., minimum, maximum, or mean trait values). Functional traits in MOSAIC may be estimated from populations studied in COMADRE, COMPADRE, PADRINO, or other databases (Table S4). The potential temporal and spatial mismatch between databases that are linked in an analysis merits close attention^63^. The studies in the MOSAIC database also include research conducted by different investigators using independent tools, technologies, sample designs, and study methods. The influence of research methods and instruments on the error values in the dataset may require additional consideration for potential bias, noise, and imprecision. Where more than one life history trait value exists for a given species, MOSAIC users will need to determine whether averaging or selective filtering to one study is most appropriate in view of the specifics of the given research question. In certain cases, trait values for a species might only be available for a single st/age and therefore may not provide a complete picture of the trait variation amongst st/ages. Users are encouraged to be cautious when contextualising the scope of representation of the values in the database and their analyses.

### Representation, variance, and Bias in MOSAIC v1.0.0

MOSAIC has the potential to help identify macroecological patterns and guide targeted experimental studies that can mechanistically examine the causes and correlates of demographic variability. MOSAIC leverages thousands of animal and plant species housed in COMPADRE, COMADRE, and PADRINO and offers promise for evaluating general hypotheses and identifying novel ones from newly discovered patterns. Despite the inductive value and generality of macroecological inference^64–66^, caution is required in inferring process and cause from trait-demographic patterns using MOSAIC. MOSAIC is a starting point and contextualising instrument, not a stand-alone tool for inferring how traits determine demography and/or how demographic processes may shape traits. In its version 1.0.0, MOSAIC contains a high degree of variance in trait values known to shape demographic outcomes across major taxonomic groups. For example, determinant growth is present for 0% of Amphibians and Birds and 100% of Bivalves, while volancy is present for 0% in Reptiles, Amphibians, and Bony Fish and 77% in Birds (Fig. 2). Animal adult biomass and plant height follow lognormal trait distributions (see Appendix S5). 11% of mammals are indeterminate growers *vs*. 54% of reptiles and 0% birds, and 95% of mammals are monogamous compared to 100% of reptiles and 20% of birds in MOSAIC. Recent studies have examined how different vital rates are explained by functional traits^67–70^. However, understanding how trait variation across taxa translates to demographically influential properties remains underdeveloped.

MOSAIC’s initial release (v1.0.0) includes records for all major regions of the globe for which we have structured population models (Fig. 3). Nevertheless, species trait values are not necessarily gathered from the same localities as population models (see *future directions* for more information on systematising spatial mismatch). This is an important consideration for users of MOSAIC (and more generally of trait-based approaches) wishing to bring together functional traits and demographic rates, as traits and vital rates are known to vary considerably within the same species across spatial scales^71–73^. Moreover, while there is at least one trait for each of these locations, the data density remains variable. Thus, records are not necessarily representative of the global spread or the full spatial scope of MOSAIC. For example, the highest complete coverage for MOSAIC traits is concentrated toward localities with the with the longest-term demographic models (see COMPADRE locations associated with MOSAIC records).

Phylogenetically, the initial release of MOSAIC is somewhat limited. Version 1.0.0 covers 300 of the 1,400 species currently available in COMPADRE, COMADRE and PADRINO. However, MOSAIC trait data are well distributed across clades (Fig. 4). While there is not a highly skewed phylogenetic concentration with respect to the existing structured population models or clustering of records into small groups across the Tree of Life, phylogenetic density of MOSAIC records remains low. Therefore, information from the MOSAIC database may be limited for a given genus or order and, as such, should be approached with caution. In future versions of MOSAIC, the phylogenetic bias is expected to diminish with more samples and stronger phylogenetic representation.

Covariance across traits is also an important source of confound in existing analyses linking traits and vital rates. Positive and negative correlations across traits that have independent influences on vital rates can create apparent associations of demographic properties with traits, spuriously functionalising non-functional traits^74^. Disentangling the relevance of key axes of trait variation for their demographic influences demands a clear quantification of the direction and strength networks of trait associations, trade-offs, and demographic consequences. Population biologists seek to understand not only how individual traits relate to different aspects of demographic performance (*e.g*., population growth rate, risk of quasi-extinction, *etc*.), but also understanding how trait syndromes shape those demographic outcomes. MOSAIC presents a highly varied covariance structure in trait values for the examined 300 species. For example, without *a priori* expectation, indeterminate growth and regeneration traits show strong correlation (r = 0.51; Fig. 5), which could influence each other’s effects on vital rates. The same could be argued for the correlation between volancy and reproductive strategy (r = 0.28 with monogamy; Fig. 5).

The MOSAIC database can be used as a platform to showcase the lack of overlap between trait and vital rate data for the same species. This picture calls for a more systematic way address global biases in ecological data quantification/collection. Even where we have complete information about species in the COMADRE, COMPADRE, and PADRINO databases, we are subject to the constraints and biases of those datasets, such as spatial bias toward high-GDP countries and the phylogenetic bias toward temperate regional perennial plants^75,76^. The compounding of error and density across datasets highlights the need to prioritize stronger representation of functional traits linked with demography. The standardized framework of MOSAIC is an ideal platform to work from to achieve this goal.

In view of potential biases introduced by low sampling density and the patchiness of cover in traits, users of the database are advised to consult the literature about the representativeness and congruency between MOSAIC data and related trait diversity within clades. Users need to be mindful of the scope of the questions that they are setting out to answer and to be aware of the influence of sample sizes and coherency or heterogeneity of traits across different taxonomic levels.

### Extensions and relevance

#### From databases to data networks

Broad aperture digitisation efforts (*e.g*., BIEN^7,45^ and TRY^23^) have helped resolve many answers to demographic questions. Examples include whether there are trait spectra and key trade-off patterns amongst functional traits and whether these are correlated with particular environments and life history strategies^77–79^, Trait-based ecology and Trait Driver Theory^80^ are indebted to such opportunity-driven research programmes. More generally, however, the trait-based ecology paradigm has failed to support clear answers to many research questions of central interest to demographers^29,81^. This limited reach of the functional trait programme coincides with a dearth of species-specific overlap across the range of functional traits that are collected by the functional trait databases.

The proliferation of databases and open data initiatives over the last two decades^82^ evidences an interest in improving both data access and data usability^18,83,84^. While existing databases standardize trait fields, collate records, and link associated metadata, existing databases often store data for simple, quantitative traits. Relatively few ecological trait databases store diverse data types (such as rate arrays, population time series, physiological rates at different structural levels, and habitat shape files) that may be associated with multidimensional, ecological study systems (but see CESTES^17^, GFBio^85^, DarwinCore^86^).

The digitisation and standardisation of *existing* data and their integration with complementary, new data presents a growing set of challenges and opportunities in ecology^87^. Efforts to gap-fill records can leverage the value of existing datasets while expediting the schedule for specific research outcomes. As trait datasets grow, the importance of targeted, gap-filling initiatives to address bias and to capitalize on existing data will also increase^88^. The value of existing records is further enhanced through improvements in the interoperability of datasets. Much of this work is done manually, at a high cost, and with little support from funding agencies^87^, and yet it has been effective at facilitating research and creating new value for old data. In recent years, initiatives have sought to improve the interoperability of datasets by guiding prospective data structure or retroactively harmonising existing datasets. These include programmes that develop universal standards to improve global interoperability (such as DarwinCore^86^ and Frictionless [https://frictionlessdata.io/]data standards) or that contain guidelines for data metastructure (such as the FAIR principles (findability, accessibility, interoperability, and reusability, *sensu* Wilkinson *et al*., 2016) and the OpenTraits framework^18^). These initiatives address emerging and scaling challenges of ecoinformatics, such as the protocols by which we share data, search data, and preserve provenance in data storage structures. These protocols will be essential in centralising datasets as diverse as government monitoring datasets (*e.g*., those stored in U.S. Data clearing houses [https://www.data.gov; https://www.dataone.org/]; National Biodiversity Atlas [https://nbnatlas.org/]); centralized monitoring and experimental networks (*e.g*., LTER and NEON), raw or reanalyzed remote sensing datasets (*e.g*., Landsat data, NASA EarthData datasets, ERA-5 data), and private datasets (https://www.natureserve.org/) that will demand versatile and navigationally efficient data structures.

Population ecology has benefitted from widespread open-access databases but requires further dataset integration to answer its central questions. Understanding whether and how some morphological or physiological traits predict demographic outcomes and why others fail to do so is of central interest to questions in physiological, population, and community ecology^29,69,89,90^. Population ecologists routinely use data that are distributed across a wide range of databases. Comparative and macroecological researchers use phylogenies^91–93^, adult bodymass^61,94–96^, and high-resolution, global climate information^97–99^ to answer relevant biological, evolutionary and ecological questions and to contextualize their findings. Population ecologists frequently examine a subset of physiological, morphological, and behavioural attributes associated with demographic outcomes (*i.e*., functional traits^100^). The trait-based research programme seeks to, among other aims^6^, identify the intrinsic and extrinsic regulators of vital rates and the causes of variation and constraints on possible trait values^65,66^. Not all traits predict demographic outcomes and functional traits may exercize influence on only a few demographic pathways^68,101^. The answers to these questions rely on the existence of vital rate and trait data, the overlap of which has been limited in the absence of targeted attention. For instance, of the hundreds of thousands of records available across thousands of plant species in TRY^23^ and over 345 plant species in COMPADRE^46^, Adler *et al*. (2014) report functional trait-vital rate relationships for only 222 plant species due to their limited data overlap.

Ecological data are complex and their structures will need consistent rules to link datasets together. It will be important for future datasets to adopt database designs that render large, thematically, and structurally diverse data to be readily locatable and usable without expert knowledge. Here, we show one such example in the scope of comparative research, using thematic groups and a strategy of achieving adequate record breadth before revisiting depth of records for specific species. The need for open access data, integrated workflows, and interoperable data systems is increasing with the scaling of data collection through use of robotics and technologies. The gaps in existing data systems, interoperability, and data acquisition can be filled strategically for specific applications, offering targeted and efficient dataset development. With data interoperability guiding the structure of new datasets, the modular development of area-specific datasets will enable more generalized use over time and help meet the aims of existing database initiatives.

## Supplementary information

Supplementary Information

## Data Availability

Convenience functions for navigating the MOSAIC database are included in the supplemental material (S5) and on the MOSAIC website https://mosaicdatabase.web.ox.ac.uk and associated GitHub repository https://github.com/mosaicdatabase/Rmosaic. All code is open access without restrictions on access.
