# Supplementary material for: MOSAIC - A Unified Trait Database to Complement Structured Population Models
Source: Sci Data. 2023 Jun 1;10:335. doi: 10.1038/s41597-023-02070-w (PMC10235418; doi:10.1038/s41597-023-02070-w)
Supplement: Supplementary file 1 — Supplementary Information [file 41597_2023_2070_MOESM1_ESM.pdf]

MOSAIC: A Unified Trait Database to Complement Structured Population Models

Connor Bernard, Gabriel Silva Santos, Jacques Deere, Roberto Rodriguez-Caro, Pol Capdevila, Erik Kusch, Samuel J L Gascoigne, John Jackson, & Roberto Salguero-Gómez

SUPPLEMENTARY ONLINE MATERIALS

Table of Contents

S1: DATABASE LICENSING TERMS FOR CONSTITUENT DATABASES OF MOSAIC 2
S2: MOSAIC USER GUIDE 3
S3: MOSAIC TRAIT PRIMARY SEARCH KEY WORDS 32
S4. DATABASES SEARCHED 34
S5: VIGNETTES 35
S6: LOGNORMAL DISTRIBUTION OF MASS AND HEIGHT 76

S1: Database Licensing Terms for Constituent Databases of MOSAIC

Database Name	Use License	Source of Licensing Terms
BIEN	CC-BY-NC-ND/CC 4.0	https://bien.nceas.ucsb.edu/bien/biendata/data-use-policy/
TRY	CC-BY	https://www.try-db.org/TryWeb/About.php
OTL	CC 4.0	https://mctavishlab.github.io/R_OpenTree_tutorials/LICENSE.html
AMNIOTE	CC 4.0	https://figshare.com/articles/poster/An_Amniote_Life_History_Database_to_Perform_Comparative_Analyses_with_Birds_Mammals_and_Reptiles/1098914
AMPHIBIO	CC BY 4.0	https://www.nature.com/articles/sdata2017123.pdf?proof=t%3B
PANTHERIA	CC0	https://figshare.com/collections/PanTHERIA_a_species-level_database_of_life_history_ecology_and_geography_of_extant_and_recently_extinct_mammals/3301274
ANAGE	CC-BY 3.0	https://genomics.senescence.info/legal.html
TREE OF SEX	CC 4.0	https://www.nature.com/articles/sdata201415
OPEN TRAITS	CC-BY max restrictiveness	Datasets listed under: https://opentraits.org/datasets.html

S2: MOSAIC User Guide

USER GUIDE TO THE MOSAIC LIFE HISTORY DATABASE

A Companion to COM(P)ADRE and PADRINO/A demographic databases

Working document – 06 February 2022

Table of Contents

User Guide Information

User Guide

Appendix

References

Table of Contents

Introduction

General Instructions

Database Organization	Page 6
Database Design	Page 6
The meanings of NA in MOSAIC	Page 6
Disclaimer	Page 6
What is new in this version?	Page 7

Format of the User Guide

Format Diagram	Page 8
Variables in MOSAIC	Page 9

Metadata

A. Species Name/Taxonomy

1.	**A1**	Species Accepted	Page 10
2.	**A2**	Kingdom	Page 11

B. Study Information

3.	**B1**	Author	Page 12
4.	**B2**	Journal Name	Page 13
5.	**B3**	Year Publication	Page 14
6.	**B4**	DOI/ISBN	Page 15

Primary User Guide/Variables

* Applicable to plants

** Applicable to animals

A. Morphometry & Growth

1.	**A1**	Biomass	Page 17
2.	**A2**	Height	Page 18
3.	**A3**	Growth Determination	Page 19
4.	**A4**	Regeneration	Page 20
5.	**A5**	Sexual Dimorphism	Page 21

B. Reproductive traits

6. **B1** Mating System** Page 22
7. **B2** Hermaphrodism Page 23
8. **B3** Protogyny/Protandry** Page 24

C. Movement traits
9. **C1** Dispersal Capability Page 25
10. **C2** Type of Dispersal Page 26
11. **C3** Mode of Dispersal Page 27
12. **C4** Dispersal Class Page 28
13. **C5** Volancy** Page 29

User guide version information

Version 1.0.0

Release date: 6 February 2021

Contact: mosaicdatabase@biology.ox.ac.uk

General Instructions

Database Organization

The data associated with MOSAIC are provided in a single R data (extension.Rdata) and as a comma separated value (.csv) file format. The code for downloading the data can be located from the mosaic portal (https://mosaicdatabase.web.ox.ac.uk/download-database) In addition, these files are accompanied by R scripts and a nexus phylogeny available in the Supplementary Information of the manuscript introducing MOSAIC, and in our GitHub repository (https://github.com/mosaicdatabase/mosaicdatabase)

MOSAIC_v_1_0_0.RData: Contains basic information regarding the source of publication, as well as ecological, biogeographic, and taxonomic details of the demographic study for each study species, the demographic information (i.e., the matrix population model) and metadata.

Database Design

In developing the MOSAIC database, we balance level of detail with accessibility. A highly detailed, comprehensive profile of life history traits for species in COM(P)ADRE and PADRINO/A, if possible to collect, would be difficult to navigate. So rather than collating a wealth of information in many different formats, we designed this dataset to highlight a smaller collection of traits in a single format which is of most interest, and expressed, by COM(P)ADRE and PADRINO/A users. In MOSAIC's future updates, we plan to add additional detail and additional fields, but we initially took a limited approach and plan to keep the design minimal. If there are life history traits, alternative formats for existing variables, or other features you would like to see added to MOSAIC, please suggest them to us at: mosaicdatabase@biology.ox.ac.uk

Understanding the diverse needs of users, we include in this guidance document additional direction on obtaining information on the variables we report in more detail. In this guide, we also highlight the scope of use, and caution against the most foreseeable abuses of data. We ask that all users approach this dataset with a caution and pay close attention to what variables and their statistical expression reflect.

The Meanings of NA and NDY in MOSAIC

NA in the MOSAIC data generally means that the data are not applicable. An example of where the data are not applicable is volancy within plants, as this trait does not occur in plants. NDY in the MOSAIC data means that the data have not yet been digitized. NF in the MOSAIC data means that the data are not available to date as no affirmative records were found upon review.

Disclaimer

The MOSAIC digitization team does its best to ensure data accuracy, and every piece of information goes through multiple error-checks prior to its release in www. mosaicdatabase.web.ox.ac.uk. However, we claim no responsibility for any damage that may arise from using MOSAIC. A list of error checks and potential issues in the use and

interpretation of the database are described in the main manuscript. The end user is ultimately responsible for his/her interpretations of the data.

What is new in this version?

Version 1.0.0

- The first version of the database. No updates.

DIAGRAM OF THE MOSAIC DATABASE ARCHITECTURE

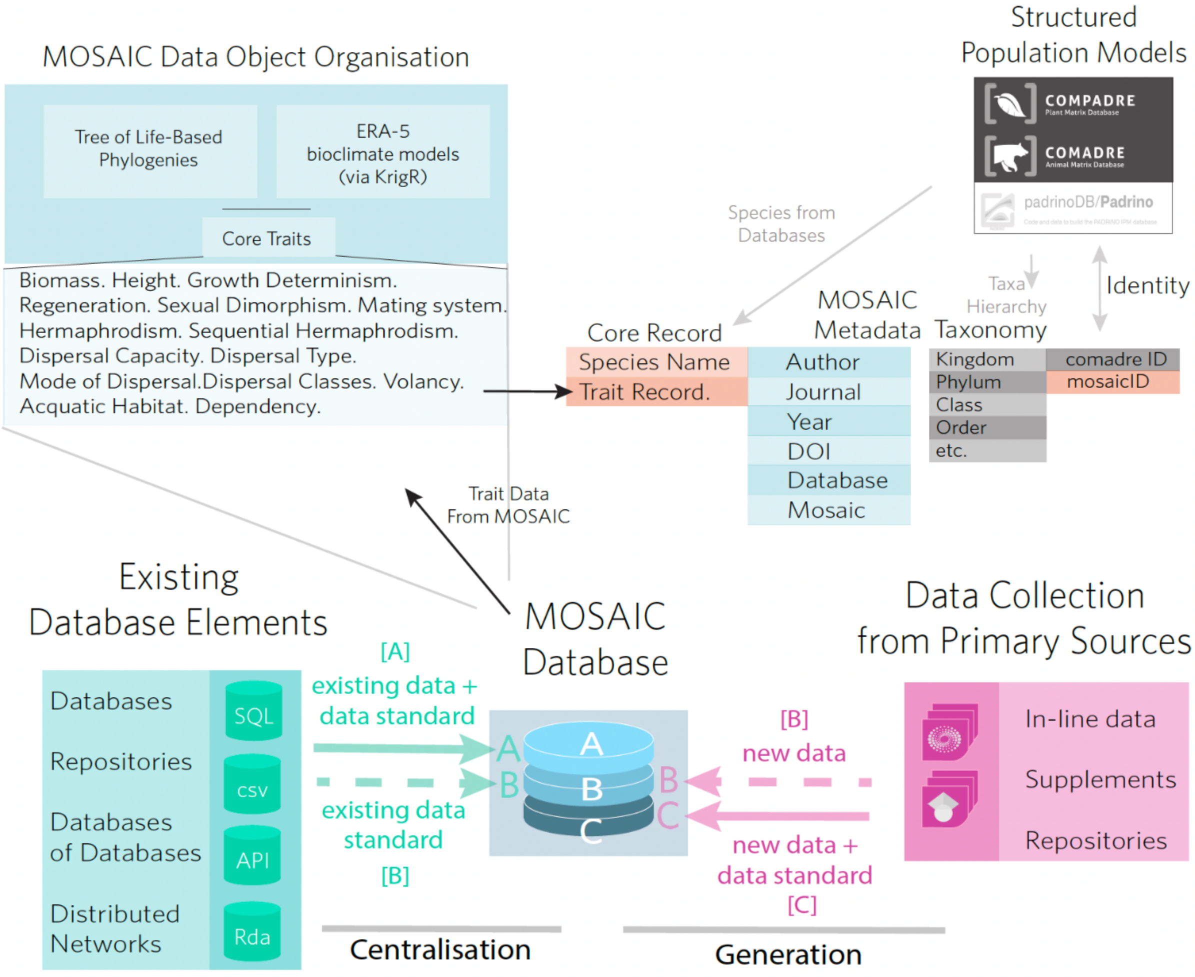

Variables in MOSAIC

The MOSAIC database is constructed of objects containing life history information across variables and organized into themes to aid in navigation. The metadata object is the object containing information about every study for which data is stored in the MOSAIC database. Every variable containing a value in the MOSAIC database will have corresponding metadata.

Associated with every data record is a value and its corresponding metadata. The metadata details data providence and relationships to existing databases. The fields within metadata are detailed below:

Format Guide

[Index] *Variable Name*

Definition: [Definition of the variable]

Possible values, [cat. = categorical/discrete; cont. = continuous], [r variable class: character, numeric, integer, complex, or logical.]

- <XX> [variable with two digits]
  XXX –XXX [discrete variable name] – [discrete variable definition]
  <...>XXX<...> [discrete variable with additional content on either side]
- Units: [unit of measure (integer, percent, ratio, mm, km^2, g, etc.)]
- Precision: [for continuous variables: scientific notation of the precision of the measure - e.g., $1e^1$ for km = precise to the tens of km (3270 km), no decimal; $1e^{-1}$ for temperature = precise to the tenth of a degree (10.4 °C)]
- Error boundaries: [for continuous variables: boundaries beyond which values are errors]

Usage Notes: [Notes on boundaries of use – note that this is non-exhaustive and highlights major potential errors]

Additional Information: [Guidance on databases containing more detailed information]

Source Data: [Datasets from which information was gathered; note that this will not include individual papers unless the papers are associated with a larger dataset/database. This category reflects databases.]

Last updated: [Date that information was retrieved from other databases or searches]

A1 *Species Accepted*

Definition: Currently accepted latin name.

Possible values, cat., factor

- <Genus_species> - e.g., Taraxacum_officinale

Usage Notes: NA

Additional Information: NA

Source Data: This information is obtained from The Encyclopaedia of Life

Last updated: 24 January 2022

A2 *Kingdom*

Definition: Kingdom to which species belongs

Possible values, cat., factor

- <kingdom> - e.g., Plantae, Fungi, Rhodophyta, Chromista (yes, MOSAIC includes fungi and algae as well as plants)

Usage Notes: NA

Additional Information: NA

Source Data: The Encyclopaedia of Life

Last updated: 24 January 2022

B1 *Authors*

Definition: Surname (family name) of all authors

Possible values, NA, character

- <name(s)> - Separated with ";" e.g., "Smith; Jones"

Usage Notes: NA

Additional Information: NA

Source Data: NA

Last updated: 24 January 2022

B2 *Journal Name*

Definition: The document from which data were sourced.

Possible values, cat., factor

- <abbreviated journal name> - Where the data come from a scientific journal article, the abbreviated journal name is given. We use the standard abbreviation of the journal compliant with the ISO-4 standard.
- Book - Records are from a book, or book chapter
- PhD thesis - Records are from a doctoral thesis

Usage Notes: NA

Additional Information: NA

Source Data: NA

Last updated: 24 January 2022

B3 *Year Publication*

Definition: Year of publication

Possible values, cont., numerical
- <yyyy> - e.g., 2012

Usage Notes: NA

Additional Information: NA

Source Data: NA

Last updated: 24 January 2022

B4 *DOI/ISBN Number*

Definition: Digital Object Identifier number

Possible values, NA, character

- <XXXXXXXXXXXXXXX> - e.g., doi.org/10.1073/pnas.1506215112

Usage Notes: NA

Additional Information: NA

Source Data: NA

Last updated: 24 January 2022

PRIMARY Variables

A1 *Biomass*

Definition: Maximum reported mass of adult individual/whole-organism. For plants, only aboveground dry mass is measured. See "Additional information" for additional information on belowground biomass.

Possible values, cont., numerical

- Units: Grams (g)
- Precision: 0.000
- Error Boundaries: 0-150,000,000g
- NF – Body mass reviewed and inconclusive (no affirmative records found upon review)
- NDY – Body mass not digitised yet (yet to be evaluated)
- NA – not applicable

Usage notes: When both male and female data were reported, only the maximum value was considered independently of the gender of the individuals.

Additional Information: Belowground biomass is not reported because information availability is appreciably more limited than for aboveground biomass. The BIEN database includes information on belowground biomass that can be referenced and utilized where of interest.

Source Data: Amniote, TRY database.

Last updated: 24 January 2022

A2 *Height*

Call: -Morph$Height

Definition: (Plants) Maximum height of the whole organism/whole individual from surface (i.e. substrate) to tallest vertical extremity.

Possible values, cont., numerical

- Units: centimeters (cm)
- Precision: 0.000
- Error Boundaries: 0-1000cm

Usage notes:

- Depending on the species group, height can have profoundly different meanings. Height corresponds with embolic risk in some woody plants and corresponds with size-based fitness in others.

Additional Information: NA

Source Data: TRY database.

Last updated: 24 January 2022

A3 *Growth Determination*

Call: -Morph$Growth$Determination

Definition: Growth indeterminacy is defined by continuous growth of individuals throughout their lifetimes (measured by mass, length, bone ossification, or other indicators). This field reflects a binary classification of whether an individual is growth (in)determinate.

Possible values, cat, factor

- Growth indeterminate – growth continues throughout an individual's lifespan
- Growth determinate – growth ceases or attenuates to negligibility before the end of an individual’s lifespan
- NF - Growth determination reviewed and inconclusive (no affirmative records found upon review)
- NDY - Growth determination not digitised yet (yet to be evaluated)
- NA – Growth determination not applicable to the subject area

Usage notes: Additional classification systems for characterizing growth indeterminacy exist in the literature. Most well recognised is a six-type scheme describing growth and determination by Sebens (1987), which offers detailed characterization of growth patterns. Broad-scale information about growth and age for most species could not be located in the literature, and therefore a simplified schema is used. More resolved classifications might be incorporated into future versions of MOSAIC.

Additional Information: NA

Source Data: 24 January 2022

Last updated: NA

A4 *Regeneration*

Call: **-**Morphology$Growth$Regeneration

Definition: Capacity for an individual to regenerate any substantial part of its body, including autotomy. Autotomy is defined as "The voluntary severance by an animal of a part of its body (commonly one of its own limbs), usually to escape capture by a predator that has seized that part. The part then regrows."

Possible values, cat., factor

- Regenerative – individuals are capable of regenerating
- Non-regenerative – individuals do not exhibit the capacity to regenerate tissues
- NF - regenerative abilities reviewed and inconclusive (no affirmative records found upon review)
- NDY - regeneration information not digitised yet (yet to be evaluated)
- NA – regenerative abilities not applicable to the subject area

Usage notes: There are a number of more resolved schemes for detailing whether regenerating different parts of the body. For particular questions pertaining to the nature of injury, the level of recovery, the role of depredation, and the consequences for reproduction, more detail may be appropriate. This dataset covers the most general applications and initially screens for regenerative capability (from poor recovery of appendages to complete regeneration of limbs).

Additional Information: NA

Source Data: NA

Last updated: 24 January 2022

A5 *Sexual Dimorphism*

Call: -Morph$Dimorph

Definition: An indicator of whether sexual dimorphism is exhibited in the species. Sexual dimorphism is defined as “the occurrence of morphological differences (other than primary sexual characters) that distinguish males from females of a species of organism.” (Oxford Dictionaries of Ecology and Zoology)

Possible values, cat., factor

- Sexually Dimorphic – species is sexually dimorphic
- Sexually Monomorphic – species is sexually monomorphic (i.e. non-dimorphic)
- NF - Sexual dimorphism reviewed and inconclusive (no affirmative records found upon review)
- NDY - Sexual dimorphism not digitised yet (yet to be evaluated)
- NA – Sexual dimorphism not applicable to the subject area

Usage Notes: NA

Additional Information:

Source Data: NA

Last updated: 24 January 2022

B1 *Mating system*

Call: **-**Reproduction$MatingSystem

Definition: System of mating; the organization of sexual interactions of individuals within populations based on sex.

Possible values, cat., factor

- Monogamy – exclusive mating between one male and one female
- Non-monogamy - Non-monogamy was assigned based on genetic or behavioural evidence
- NF - Mating system reviewed and inconclusive (no affirmative records found upon review)
- NDY - Mating system not digitised yet (yet to be evaluated)
- NA – Mating system not applicable to the subject area

Usage notes:

- Metric does not identify size of groups for plural mating systems
- Metric does not identify enforcement mechanisms for different mating systems

Additional Information: NA

Source Data: NA

Last updated: 24 January 2022

B2 *Sexual allocation*

Call: **-**Reproduction$Allocation

Definition: Indicator of whether a species exhibits hermaphroditism or monoeicieosity. Hermaphrodism is defined as: “An individual that possesses both male and female sex organs; i.e. it is bisexual.” (Oxford Dictionary of Zoology). Monoeciousness is defined as: “Applied to an organism in which separate male and female organs occur on the same individual (e.g. to a plant which bears male and female reproductive structures in the same flower or separate male and female flowers on the same plant, or to a hermaphrodite animal). Some authors restrict the term botanically to plants with separate male and female flowers; plants which bear male and female reproductive organs in the same flower are then called hermaphrodite.”

Possible values, cat., factor
- Hermaphroditic – species is hermaphroditic
- Monoecious – species is monoecious
- Dioecious or Gonochorous – species is dioecious (Gonochorous was adopted for animals)
- NF - Hermaphroditism reviewed and inconclusive (no affirmative records found upon review)
- NDY - Hermaphroditism not digitised yet (yet to be evaluated)
- NA – Hermaphroditism is not applicable to the subject area

Usage notes:
-

Additional Information: NA

Source Data: NA

Last updated: 24 January 2022

B3 *Sequesntial hermaphroditism*

Call: Reproduction$SeqHermaph

Definition: Indicator of whether there is a sex switch during the organism's lifespan.

Possible values, cat., factor

- Protogynous – species is protogynous: organisms that are female and at some point in their lifespan change sex to male.
- Protandrous – species is protandrous: organisms that are male and at some point in their lifespan change sex to male.
- NF - Protogyny/Protandry reviewed and inconclusive (no affirmative records found upon review)
- NDY - Protogyny/Protandry not digitised yet (yet to be evaluated)
- NA – Protogyny/Protandry is not applicable to the subject area.

Usage notes: NA

Additional Information: Note that is not rare flowers present protogyny/protandry but this was not considered in Mosaic so far

Source Data: NA

Last updated: 24 January 2022

C1 *Dispersal Capability*

Call: -Movement$Dispersal

Definition: An indicator for whether or not a species exhibits dispersal behaviour or at any stage in its life cycle. Where dispersing, a categorical description of whether dispersal is natal or breeding or otherwise. Dispersal is defined as “The tendency of an organism to move away, either from its birth site (natal dispersal) or breeding site (breeding dispersal): the opposite of philopatry.” (Oxford Dictionary of Zoology).

Possible values: cat., factor
- Dispersing – Exhibits at least one age-/stage-class which disperses; natal or breeding components unknown.
- Natal Dispersal – Permanent dispersal of at least one age-/stage-class
- Breeding Dispersal – Dispersal of adults between breeding attempts in at least one age-/stage-class
- Multi-Dispersal – Both natal and breeding dispersal reported in the species; see DispClasses for more information
- Non-Dispersing – Species observed to have no dispersal traits/behaviour
- NF - dispersal capability reviewed and inconclusive (no affirmative records found upon review)
- NDY – dispersal capability unknown/not yet evaluated
- NA – not applicable

Usage Notes: NA

Additional Information: NA

Source Data: NA

Last updated: 24 January 2022

C2 *Type of Dispersal*

Call: -Movement$TypeDisp

Definition: An indication of whether dispersal is a passive (requires assistance) or active (no assistance) event. See DispClasses for more information.

Possible values: cat., factor

- Active – organism utilises its own morphology for the dispersal event
- Passive – organism is unable to disperse through their own means and require an external factor
- Active and Passive – organism is able to disperse with assistance but can also use external factors. Active and passive dispersal can occur within the same life stage or can occur in different life stages.
- NF – type of dispersal reviewed and inconclusive (no affirmative records found upon review)
- NDY – type of dispersal unknown/not yet evaluated
- NA – not applicable

Usage Notes: NA

Additional Information: NA

Source Data: NA

Last updated: 24 January 2022

C3 *Mode of Dispersal*

Call: -Movement$ModeDisp

Definition: An indicator of the mode of dispersal of the species (plant and animal specific terminology).

Possible values: cat., factor

- Motile – the dispersal of animal species without assistance
- Phoretic – the dispersal of animal species by attaching to another animal
- Water currents – the dispersal of animal species by water
- Motile and water currents – animal species that disperse without assistance and by water, both forms of dispersal can occur within the same life stage or can occur in different life stages
- Anemochory – the dispersal of plant seeds by wind
- Anthropochory – the dispersal of plant seeds by humans
- Autochory – the dispersal of plant seeds without assistance from an external vector (e.g., by gravity or ballistic dispersal)
- Hydrochory – the dispersal of plant seeds by water
- Zoochory – the dispersal of plant seeds by animals
- Anemochory and Anthropochory – plant seeds can be dispersed by wind and humans
- Anemochory and Autochory – plant seeds can be dispersed by wind and without the help of an external vector
- Anemochory and Hydrochory – plant seeds can be dispersed by wind and water
- Anemochory and Zoochory – plant seeds can be dispersed by wind and animals
- Autochory and Hydrochory – plant seeds can be dispersed without the help of an external vector and by water
- Autochory and Zoochory – plant seeds can be dispersed without the help of an external vector and by animals
- Hydrochory and Zoochory – plant seeds can be dispersed by water and animals
- Autochory, Anthropochory and Zoochory – plant seeds can be dispersed without the help of an external vector, by humans and by animals
- NF – mode of dispersal reviewed and inconclusive (no affirmative records found upon review)
- NDY – mode of dispersal unknown/not yet evaluated
- NA – not applicable

Usage Notes: Plant seed dispersal modes can be subdivided into further categories, however we collated lower order categories into the higher order categories identified here

Additional Information: NA

Source Data: NA

Last updated: 24 January 2022

C4 *Dispersal Class*

Call: -Movement$DispClass

Definition: Age- or stage-classes of the species that are capable of dispersal.

Possible values: cat., factor

- Adult – dispersal stage is an individual that has reached maturity, we include sub-adults into this category
- Egg – dispersal stage is a vessel within which an embryo develops and is expelled by an adult allowing for dispersal
- Fertile material – dispersal stage is a part of an individual, or in some cases a complete individual, that contains fertile material (e.g., the alga *Fucus vesiculosus*; detached floating material/individual that contains gametes)
- Gamete – dispersal stage is the reproductive cell not within a vessel
- Juvenile – dispersal stage is an individual that has not reached maturity
- Larval – dispersal in a specific juvenile stage restricted to non-mammal species, species can have multiple larval stages
- Seed – dispersal stage is fertilized, specific to plant species and, in our definition, references seeds and/or fruits that are dispersed
- Sperm – dispersal stage is the male gamete
- Spore – dispersal stage is a single cell that only contains half of the chromosome of the adult, can produce eggs or sperm
- Sporophyte – dispersal stage is a nonsexual phase of a species producing two diploid spores
- Zoospore – dispersal stage is a motile asexual spore
- Zygote – dispersal stage is a fused male and female gamete
- Adult and Juvenile – dispersal stage can be both the adult and juvenile stage
- Egg and Larval – dispersal stage can be both the egg and larval stage
- Gamete and Spore – dispersal stage can be both the gamete and spore
- Zoospore, Sperm and Sporophyte – dispersal stage can be a zoospore, sperm or sporophyte
- NF – dispersal class reviewed and inconclusive (no affirmative records found upon review)
- NDY – dispersal class unknown/not yet evaluated
- NA – not applicable

Usage Notes: Dispersal can occur in more than one age- or stage- class, where this occurs it is noted as such within the database

Additional Information: NA

Source Data: NA

Last updated: 24 January 2022

C5 *Volancy*

Call: -Movement$Volancy

Definition: An indicator of whether a species is volant or non-volant (i.e., able to fly or not).

Possible values, cat., factor

- Volant – the species is volant
  (Most Birds (Class Aves), all Bats (Order Chiroptera), and some invertebrate species)
- Non-volant – the species is non-volant
- Semi-volant – the species has gliding abilities
  (e.g., Gliding lizards (*Draco* spp.); flying squirrels such as the Northern flying squirrel (*Glaucomys sabrinus*); flying fish (Exocoetidae); gliding frogs such as Wallace's flying frog (*Rhacophorus nigropalmatus*); and gliding ants such as *Cephalotes atratus*).
- NF – volancy reviewed and inconclusive (no affirmative records found upon review)
- NDY – volancy unknown/not yet evaluated
- NA – not applicable

Usage Notes: NA

Additional Information: NA

Source Data: NA

Last updated: 24 January 2022

S3: MOSAIC Trait Primary Search Key Words

MOSAIC: Keywords used in primary literature search

Taxonomic names (binomial nomenclature) were used in connection with the field-specific terms in the search of primary literature in identifying the records of interest. The below include names that were searched in review of the primary literature.

Biomass—
NA (database only)

Height—
NA (database only)

Growth determination—
Sources: *Web of Science; Google Scholar; Scopus*
Terms: Growth determinat*, growth determination, growth determinate, growth indetermination, growth

Regeneration—
Sources: *Web of Science; Google Scholar; Scopus*
Terms: Regenerat*, regeneration, regenerate, rejuvenation

Sexual dimorphism—
Sources: *Web of Science; Google Scholar; Scopus*
Terms: Dimorphic

Mating system—
Sources: *Web of Science; Google Scholar; Scopus*
Terms: Monogamy, monogamous, polygyny, polygynous, polyandry, polyandrous, mating system

Hermaphrodism—
Mating system—
Sources: *Web of Science; Google Scholar; Scopus*
Terms: Hermaphrod*, hermaphroditic, hermaphrodism, hermaphrodite, gonochoric, gynochorous, monoecious, dioecious, sexual differentiation,

Sequential hermaphrodism—
Sources: *Web of Science; Google Scholar; Scopus*
Terms: Sequential hermaphrodism, protandry, protogyny, protogynous hermaphroditism, protandrous hermaphroditism

Dispersal capacity—

Sources: *Web of Science; Google Scholar; Scopus*
Terms: Dispers*, dispersal, dispersing, dispersal capacity, dispersal capability

Type of dispersal—
Sources: *Web of Science; Google Scholar; Scopus*
Terms: Dispers*, active, passive

Mode of dispersal—
Sources: *Web of Science; Google Scholar; Scopus*
Terms: Dispers*, motile, phoretic, currents, anemochory, anthropochory, autochory, hydrochory, zoochory, anthropochory

Dispersal classes—
Sources: *Web of Science; Google Scholar; Scopus*
Terms: Dispers*, adult, jouvenile, egg, fertile, seed, sperm, spore, sporophyte, zoospore, zygote, gamete

Volancy—
Sources: *Web of Science; Google Scholar; Scopus*
Terms: Volant, non-volant, flight, flying, flightless, non-flying, winged, glide, gliding, ground

Aquatic habitat dependency—
Sources: *Web of Science; Google Scholar; Scopus*
Terms: Anadromous, catandromous, estuarine, brackish, lotic, lentic, lemnitic, littoral, palagic, marine, freshwater, saltwater, sea, ocean.

S4. Databases Searched

MOSAIC: Databases Reviewed to Date for the MOSAIC Database

Type. Database Name. DOIs.

1. **Plants**. Botanical Information and Ecology Network (BIEN) (doi: 10.7287/peerj.preprints.2615v2)
2. **Plants**. TRY: Global Plant Trait Database (TRY) (doi: 10.1111/j.1365-2486.2011.02451.x)
3. **Phylogeny.** Open Tree of Life: A synthesis of phylogeny and taxonomy into a comprehensive tree of life (doi: 10.1073/pnas.1423041112)
4. **Vertebrates**. Amniote: An amniote life history database to perform comparative analysis with birds, mammals, and reptiles (doi: 10.1890/15-0846R.1)
5. **Amphibians**. AmphiBIO: a global database for amphibian ecological traits. (doi:L 10.1038/sdata.2017.123)
6. **Mammals**. PanTHERIA: a species-level database of life history, ecology, and geography of extant and recently extinct mammals (doi: 10.1890/08-1494.1)
7. **Animals**. AnAge Database of Animal Ageing and Longevity. (doi: 10.1111/j.1474-9726.2008.00442.x)
8. **General (Sex)**. Tree of Sex: A database of sexual systems (10.1038/sdata.2014.15)
9. **General (Variable)**. Open Trait Network Databases (https://opentraits.org/)

S5: Vignettes

Navigating MOSAIC

Vignette #1 - Navigating MOSAIC

Welcome to the **MOSAIC database**, a database of functional traits for comparative demography. The database, user, guide, and additional information can be found and http://mosaicdatabase.web.ox.ac.uk.

MOSAIC is a database that aggregates existing databases and adds new records for functional traits that currently do not have a database established. In this vignette, we will show you how to download the dataset, search records, and relate MOSAIC records with COMADRE, COMPADRE, and PADRINO databases.

Optional clearance of working space.

```
rm(list = ls()) # Clear your environment
if(!is.null(dev.list())) dev.off() # Clear plots/graphics
cat("\014") # Clear console
```

Downloading MOSAIC

MOSAIC can be downloaded as an S4 object by running the below code in R. S4 data objects in R are an object oriented system in the R language that allow control of constituent data fields Similar to S3 objects (which use the "$" operator). S4 are comprised of objects that can be searched with the "@" operator or slots, discussed in more detail below.

```
library(devtools) # Compulsory package to pull down packages from GitHub. Ins
tall if necessary.
install_github("mosaicdatabase/Rmosaic")
source_url("https://raw.githubusercontent.com/mosaicdatabase/mosaicdatabase/m
ain/mosaic_fetch.R") # Link to GitHub repo
mosaic <- mos_fetch("v1.0.0") # Download version 1.0.0 (active version Feb 20
22)
library(Rmosaic)
```

Basics of manually navigating MOSAIC

Mosaic traits can be searched using the "@" operator. Attribute names searched this way are analogous to the columns of a dataframe in a relational database structure.

Once downloaded, you should be able to type statements mosaicdatabase@[insertfield] (where [insertfield] is a particular trait). If you are working in Rstudio, after the “@” a drop-down of the slots (traits) should autopopulate.

```
# Three examples of querying traits (easiest for navigation)
mosaic@biomass
mosaic@height
mosaic@volancy
```

The species corresponding with each index can be queried by prompting:

```
# Three examples of querying traits (easiest for navigation)
mosaic@species
```

Data in mosaic can also be access using slots. Slots are the recommended mode of searching the database - though it has the disadvantage of not enabling the autopopulation of the attributes contained in the database (traits must be spelled out manually).

```
# You can also search these by slot (recommended)
slot(mosaic, "biomass")
slot(mosaic, "height")
slot(mosaic, "volancy")
```

Within each trait object, there are eight fields in mosaic. The first of field is called “values” and contains the data. Values are unitless values (either numeric or factorial) that are reported in units described in the **Mosaic User Guide** http://mosaicdatabase.web.ox.ac.uk/user-guide. The metadata is organised into additional attributes, reflecting the individual elements of the metadata for a given record, including the authors, journal, year of publication, databases from which data are sourced (if applicable)

mosaic@metaTaxa maps the complete taxanomic classification structure of a species - from Kingdom to species for taxonomic clustering.

The other six attributes - “author”, “year”, “journal,”doi“,”database“, and”mosaic" - are metadata corresponding with each value record. For instance:

```
mosaic@species[[2]] # For this species, let us look at volancy (flight capaci
ty) value
mosaic@volancy@value[[2]] # or, equivalently:
slot(slot(mosaic, "volancy"), "value")[[2]]
```

Corresponds with the following metadata

```
mosaic@volancy@author[[2]] # for "Acinonyx jubatus"
```

```
slot(slot(mosaic, "volancy"), "author")[[2]] # Author of the source publicati
on
slot(slot(mosaic, "volancy"), "year")[[2]] # And year of the source publicati
on
slot(slot(mosaic, "volancy"), "journal")[[2]] # The journal of the source pub
lication
#etc.
```

Using MOSAIC functions to quickly access files

A series of convenience functions can be sourced from the MOSAIC GitHub page to facilitate navigating and working with the mosaic database that can be accessed by running the follwing script.

```
source_url("https://raw.githubusercontent.com/mosaicdatabase/mosaicdatabase/m
ain/navMosaic_46.R")
```

Below we highlight some of the basic queries for which the mosaic functions can assist.

Is a species included in Mosaic?

```
spp_check("Fritillaria biflora")
## [1] TRUE
spp_check("Pagophilus groenlandicus")
## [1] FALSE
```

Can I see all records for a given trait?

```
traitAllSpp("biomass") # Only the first five records are shown for space
## [1] "NDY"    "50578"  "ND"     "52500"  "351000" "62000"
```

Can I see an overview of all records for a given species?

```
singSppTraitSummary("Aepyceros melampus")
##   biomass height   growthdet regen   dimorph         matsyst     hermaph se
qherm
## 1   52500    NDY Determinate   NDY Dimorphic Non-monogamous Gonochorous
NDY
##           dispcap disptype modedisp dispclass    volancy
## 1 Natal Dispersal   Active   Motile     Adult Non-volant
```

```
##                                   aquadep
## 1 Terrestrial, Water Habitat Independent
```

Can I see all records for more than one species?

```
sppAllTrait(c("Acinonyx jubatus", # you can also pass lists or dataframes to
this command
               "Acropora downingi",
               "Aepyceros melampus",
               "Alces alces",
               "Alligator mississippiensis"))
##                    sppnames biomass height   growthdet        regen   dim
orph
## 1           Acinonyx jubatus   50578    NDY Determinate          NDY Dimor
phic
## 2          Acropora downingi      ND    NDY         NDY Regenerative
NDY
## 3         Aepyceros melampus   52500    NDY Determinate          NDY Dimor
phic
## 4                Alces alces  351000    NDY Determinate          NDY Dimor
phic
## 5 Alligator mississippiensis   62000    NDY Determinate Regenerative
NDY
##          matsyst        hermaph seqherm         dispcap disptype       mod
edisp
## 1 Non-monogamous    Gonochorous     NDY Natal Dispersal   Active         M
otile
## 2            NDY Hermaphroditic     NDY Natal Dispersal  Passive Water cur
rents
## 3 Non-monogamous    Gonochorous     NDY Natal Dispersal   Active         M
otile
## 4 Non-monogamous    Gonochorous     NDY Natal Dispersal   Active         M
otile
## 5 Non-monogamous    Gonochorous     NDY Natal Dispersal   Active         M
otile
##           dispclass    volancy                                      aqua
dep
## 1          Juvenile Non-volant        Terrestrial, Water Habitat Independ
ent
## 2          Juvenile Non-volant                                       Mar
ine
```

```
## 3               Adult Non-volant        Terrestrial, Water Habitat Independ
ent
## 4            Juvenile Non-volant Terrestrial, Facultative Freshwater Depend
ent
## 5 Adult and Juvenile Non-volant  Terrestrial, Obligative Freshwater Depend
ent
```

Can I get a breakdown of counts/frequency of trait values?

```
traitFrequency("volancy")
##              counts  freq
## NDY            1329    NA
## Non-volant       82 0.882
## Semi-volant       1 0.011
## Volant           10 0.108
traitFrequency("growthdet")
##                counts  freq
## Determinate        31 0.596
## Indeterminate      21 0.404
## NDY              1370    NA
traitFrequency("hermaph")
##                               counts  freq
## Dioecious                         24 0.108
## Gonochorous                      106 0.475
## Hermaphroditic                    72 0.323
## Hermaphroditic & Gonochorous       1 0.004
## Monoecious                        20 0.090
## NDY                             1199    NA
```

Can I get all metadata for one or more traits?

```
metadata("volancy", 14)
##               author year                    journal database mosaic
## 1 Campos, Z. et al. 2006 The Herpetological Journal      NDY    NDY
multiMetaRecords("volancy", c(14:20))
##                                       author year
```

```
## 1                 Ekerna, L. S. & Cords, M. 2007
## 2                   Zimmerman, S. J. et al. 2019
## 3 Jack, K. M., Sheller, C. & Fedigan, L. M. 2012
## 4                                        DFO 2013
## 5                      Torres, R. T. et al. 2017
## 6                                        NDY  NDY
## 7                         Campos, Z. et al. 2006
##
journal
## 1                                                                             A
nimal Behaviour
## 2
The Condor
## 3                                                              American Journal
of Primatology
## 4 Canadian Science Advisory Secretariat Central and Arctic Region Science
Advisory Report
## 5
Oryx
## 6
NDY
## 7                                                                   The Herpeto
logical Journal
##   database mosaic
## 1      NDY    NDY
## 2      NDY    NDY
## 3      NDY    NDY
## 4      NDY    NDY
## 5      NDY    NDY
## 6      NDY    NDY
## 7      NDY    NDY
```

Tidyverse + MOSAIC

http://mosaicdatabase.web.ox.ac.uk

Updated 14 March 2022

Vignette #2 – Tidyverse + MOSAIC

MOSAIC was built for use and integration with other datasets for comparative demography. Demographers are often interested in how traits scale across different taxonomic levels. The Tidyverse https://www.tidyverse.org/ collection of R packages (Dplyr in particular) are commonly used by ecologists to simplify workflows that subset and manipulate data. Here, we walk throught the basics of how Tidy tools can be use with the MOSAIC database to quickly isolate specific groups of organism or classes of records.

```
library(tidyverse)
```

Accessing MOSAIC

Download MOSAIC from the mosaic portal. For more information on the basics of downloading MOSAIC and navigating the data structure, see: Vignette #1:

```
remotes::install_github("mosaicdatabase/Rmosaic")
library(Rmosaic)
mosaic <- mos_fetch("v1.0.0")
```

Using Tidyverse with MOSAIC

Tidyverse allows for the easily filtering of outliers and NAs, and the isolation of records to particular groups of taxa in MOSAIC. For example, one can isolate the differences in biomass between birds and mammals and see how the decomposition of variance in biomass between the groups informs the representation of biomass values in MOSAIC

```
# Bind the ID field, species, taxa, and biomass fields into a unified dataframe

biomass_table <- cbind(mosaic@mosaicID,
                       mosaic@species,
                       mosaic@taxaMetadat,
```

```
                        mosaic@biomass@value)
# Relabel the biomass field
names(biomass_table)[length(biomass_table)] <- "biomass"
# Coerce the field into a numeric values - transforms "NDY" into "NA"
biomass_table$biomass <- as.numeric(biomass_table$biomass)
# Removing an outlier and filtering out NAs
biomass_all <- biomass_table %>%
  drop_na(biomass) %>%
  filter(biomass < 12000000)
# Filter to mammals
biomass_mamm <- biomass_table %>%
  filter(Class == "Mammalia") %>%
  drop_na(biomass) # Remove results that are NA
# Filter to birds
biomass_aves <- biomass_table %>%
  filter(Class == "Aves") %>%
  drop_na(biomass)
# Plot all biomass
plot(biomass_all$`mosaic@mosaicID`, log(biomass_all$biomass), pch=16,
     xlab = "MOSAIC ID index", ylab = "Log Biomass", main = "Biomass")
# Highlight points for mammals
```

```
points(biomass_mamm$`mosaic@mosaicID`, log(biomass_mamm$biomass), pch=16, col
="red")

legend(2085, 16, legend=c("Mammals", "All Classes"),
       col=c("red", "black"), lty=1, cex=0.8)
```

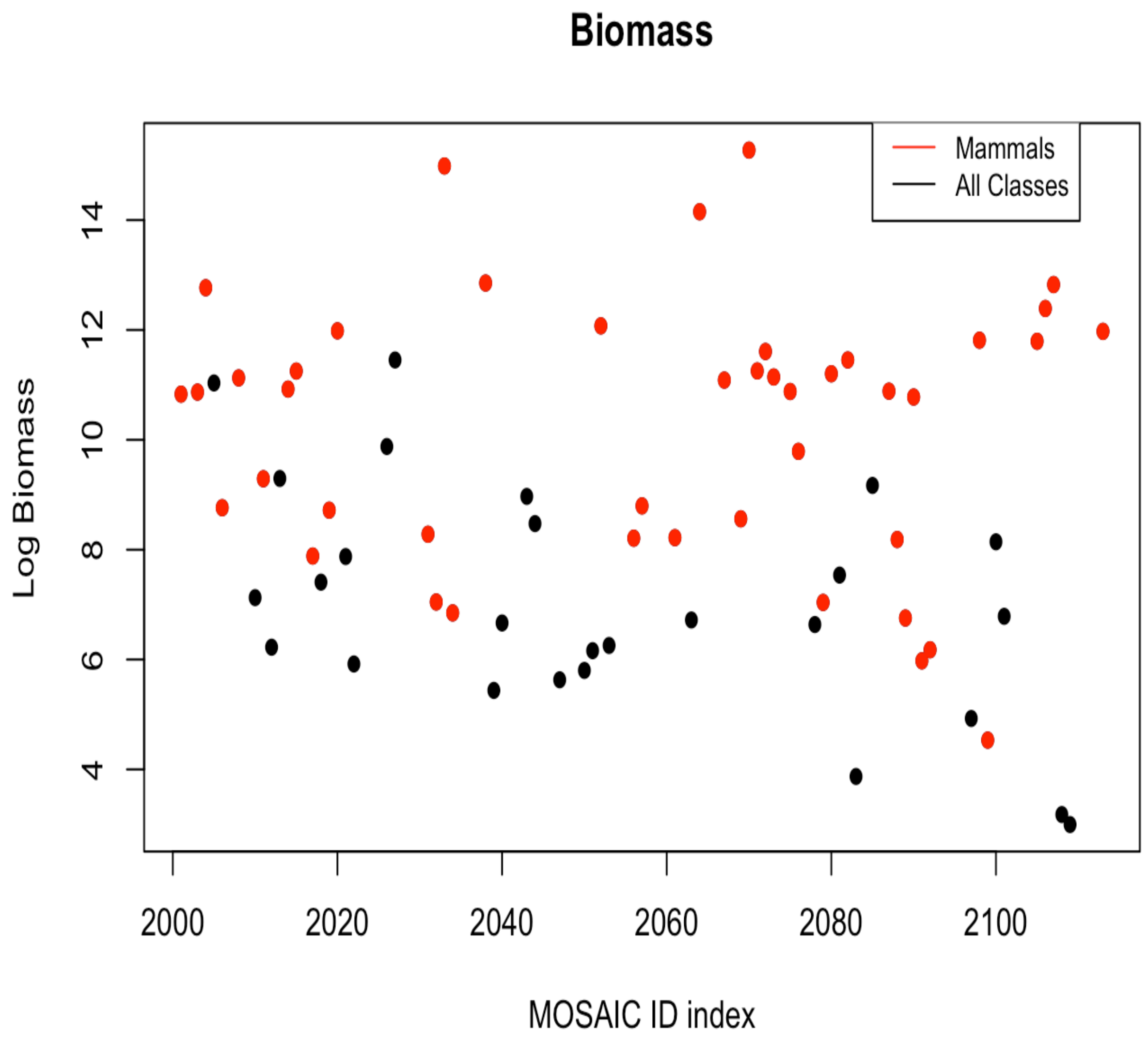


```
# Generate histograms for all species on the same binning scale

Bin_all <- hist(log(biomass_all$biomass),
```

```
                breaks=seq(min(log(biomass_all$biomass)), max(log(biomass_all
$biomass)),
                           length=15), main="Biomass (All Species)")
```

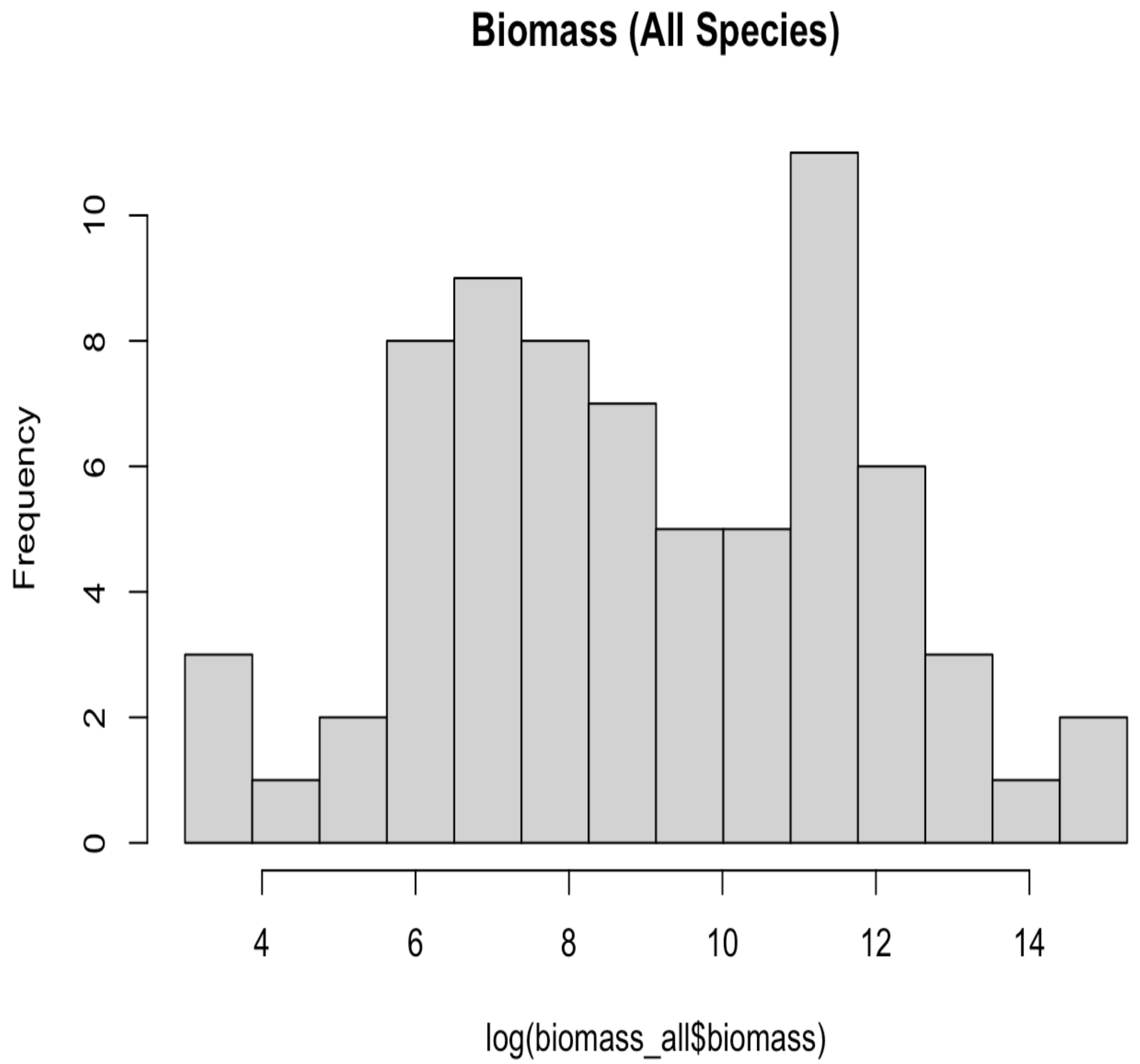


```
Bin_mamm <- hist(log(biomass_mamm$biomass),
                breaks=seq(min(log(biomass_all$biomass)), max(log(biomass_all
$biomass)),
                           length=15), main = "Mammal Biomass")
```

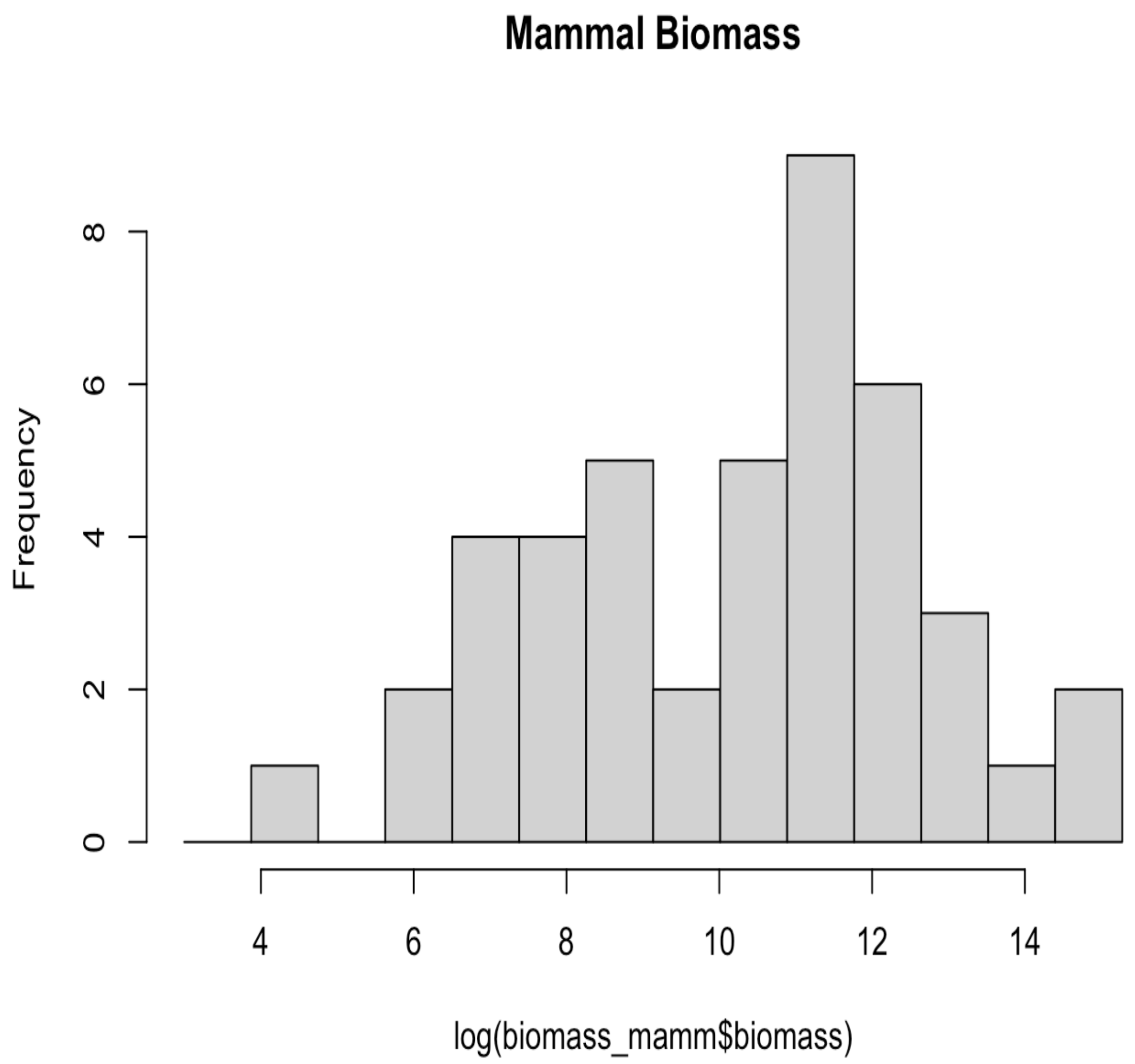


```
Bin_aves <- hist(log(biomass_aves$biomass),
                 breaks=seq(min(log(biomass_all$biomass)), max(log(biomass_all$biomass)),
                            length=15), main = "Aves Biomass")
```

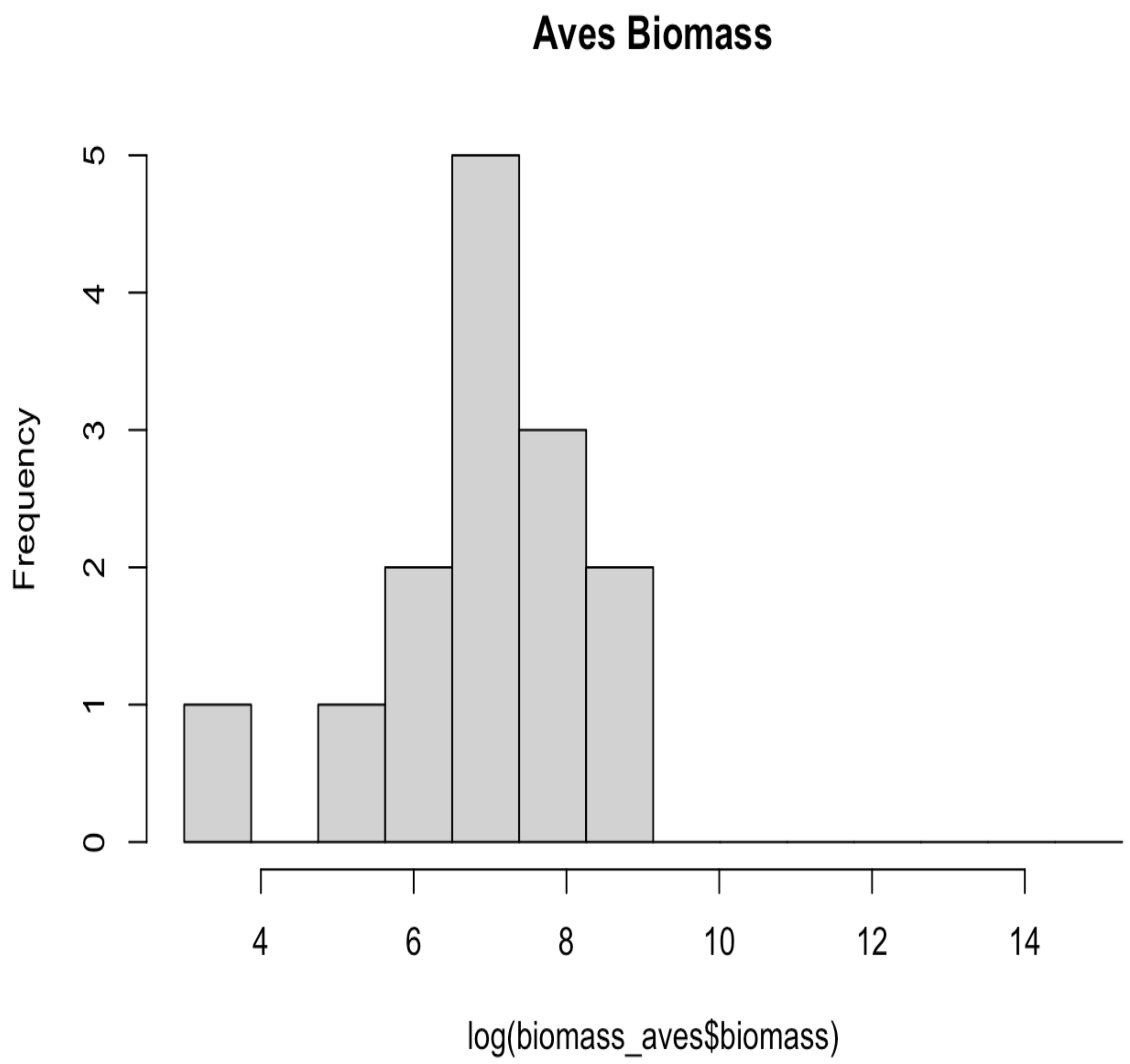


```
# Ploting frequency-density curves for species

plot(Bin_all$mids, Bin_all$counts, pch=16, type="l", col=alpha("black", 0.5), lwd=2, lty=2,
     ylim=c(0,12), ylab="record frequency", xlab="log biomass", main="Biomass by Class")
points(Bin_mamm$mids, Bin_mamm$counts, pch=16, type="l", col=alpha("red", 0.5), lwd=2)
points(Bin_aves$mids, Bin_aves$counts, pch=16, type="l", col=alpha("blue", 0.5), lwd=3)
legend(12, 12.5, legend=c("Mammals", "Birds", "All Classes"),
```

```
        col=c("red", "blue", "gray"), lty=1:2, cex=0.8)
```

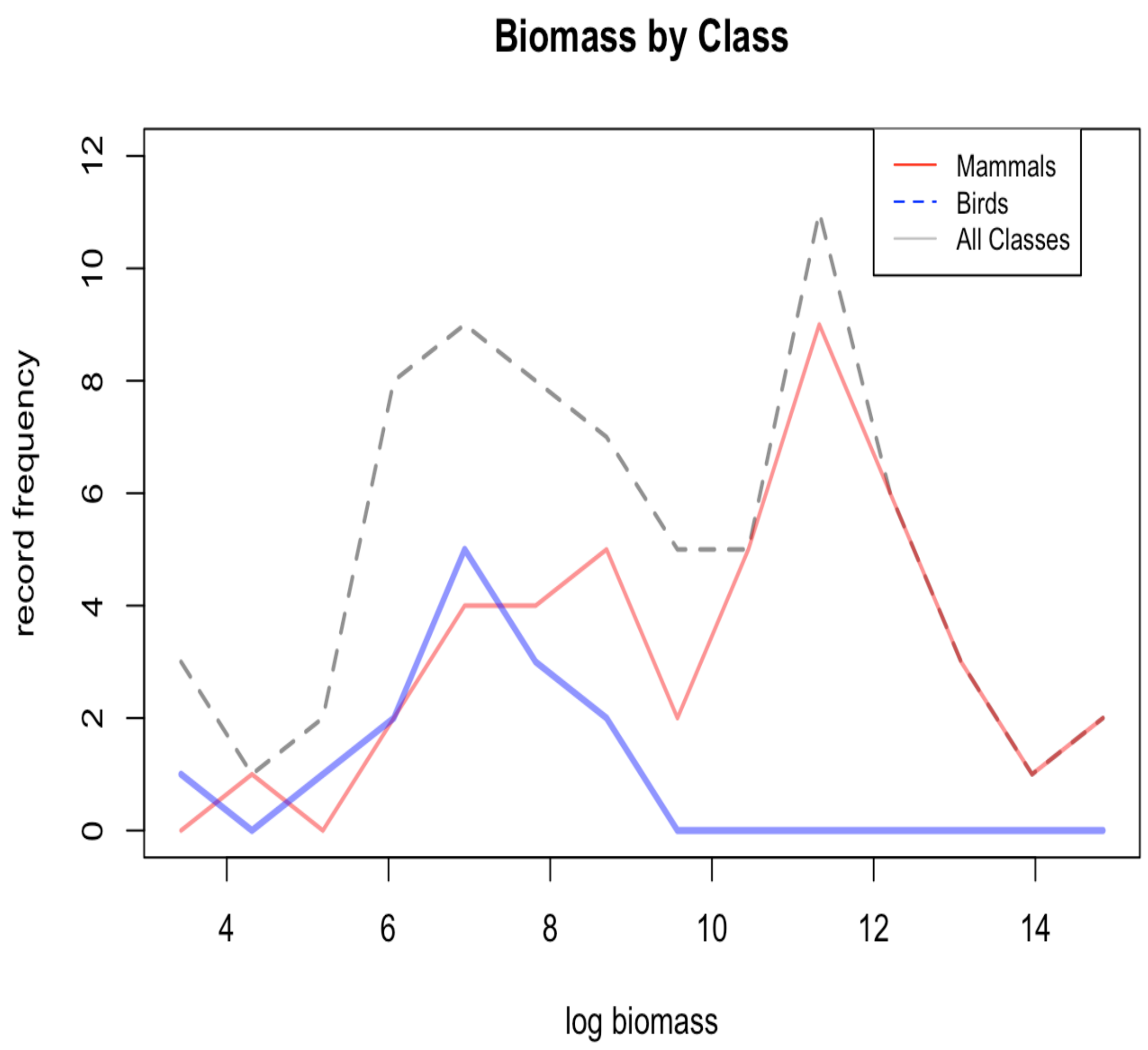


```
# Note how the peak in records of large mammals is driven by mammals, whereas
the high record count in smaller values is sustained on the aves records. Ind
ividually, the biomass distributions are semi-normal, whereas the joint distr
ibution is more complex.
```

Alternatively, the same data could be plotted by group with boxplots, illustrating the same sotry.

```
bio_mamm.aves <- cbind(log(biomass_all$biomass), log(biomass_mamm$biomass), l
og(biomass_aves$biomass))

colnames(bio_mamm.aves) <- c("all", "mammals", "birds")
```

```
boxplot(bio_mamm.aves, ylab="log biomass", xlab="class", main="Biomass by Cla
ss")
```

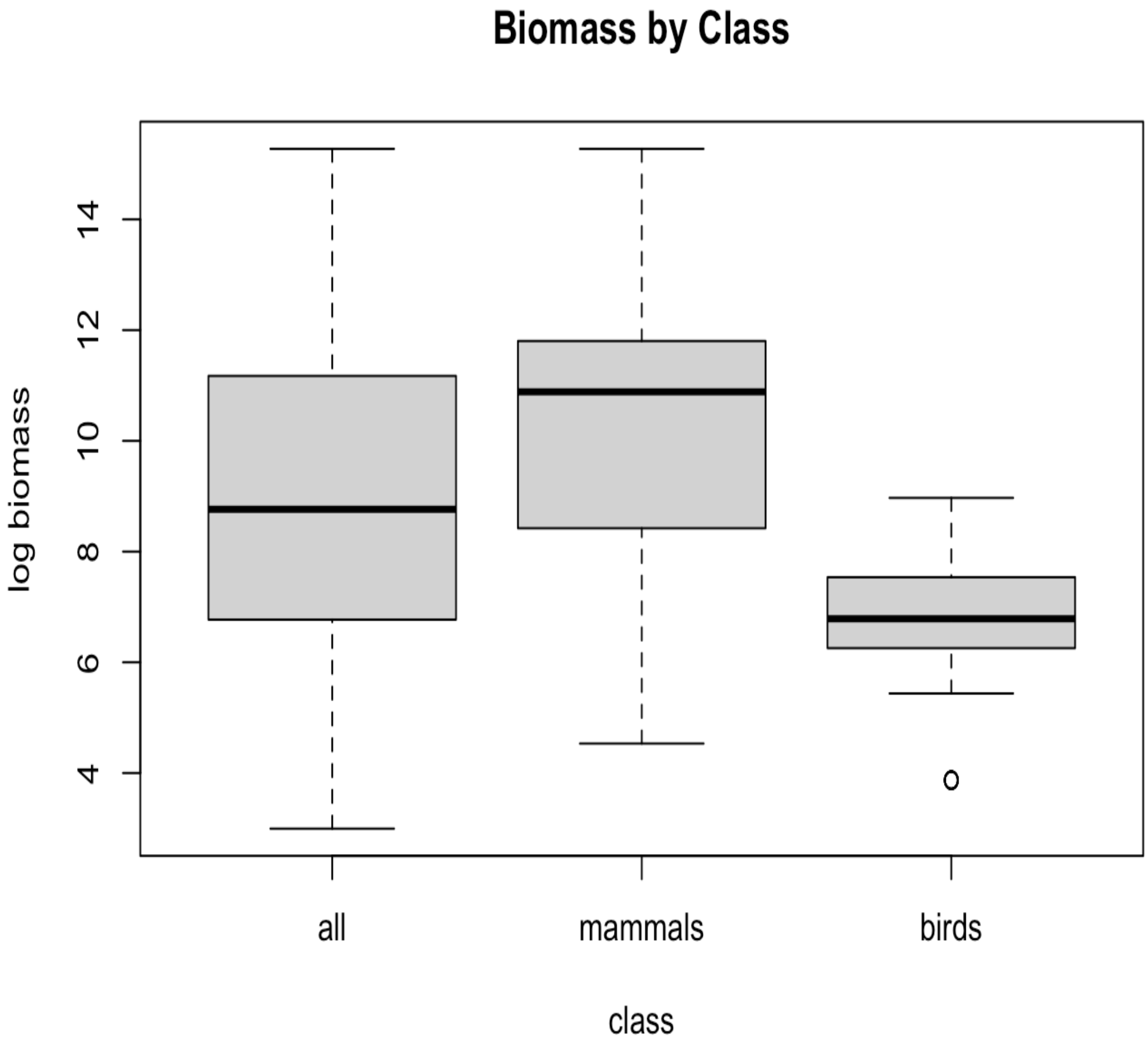

Factor variables can similarly be parsed by class.

```
# Bind the ID field, species, taxa, and volancy fields into a unified datafra
me

bio_vol <- cbind(mosaic@mosaicID,
                 mosaic@species,
                 mosaic@taxaMetadat,
                 mosaic@volancy@value)
```

```
# Relabel the volancy field

names(bio_vol)[length(bio_vol)] <- "volancy"

# Assign into a factor class

bio_vol$volancy <- as.factor(bio_vol$volancy)

# Filter out NDYs

vol_all <- bio_vol %>%
  filter(!volancy == "NDY")

# Filter to Mammals

vol_mamm <- bio_vol %>%
  filter(Class == "Mammalia") %>%
  filter(!volancy == "NDY")

# Filter to birds

vol_aves <- bio_vol %>%
  filter(Class == "Aves") %>%
  filter(!volancy == "NDY")

# Look at the summary of factors to counts

summary(vol_mamm$volancy)
##         NDY  Non-volant Semi-volant      Volant
##           0          37           1           0
summary(vol_aves$volancy)
##         NDY  Non-volant Semi-volant      Volant
##           0           3           0          10
```

```
# Store summaries

vol_all_summary <- summary(vol_all$volancy)
vol_mamm_summary <- summary(vol_mamm$volancy)
vol_aves_summary <- summary(vol_aves$volancy)

# Plot the points

plot(vol_all_summary[2:4],  type="b", pch=16, col=alpha("black", 0.5),
     ylab="record count", xlab="Volancy (1 = Nonvolant; 2 = Semivolant; 3 = Volant)", main="Volancy by Class")
points(vol_mamm_summary[2:4], type="b", pch=16, col=alpha("red", 0.5))
points(vol_aves_summary[2:4], type="b", pch=16, col=alpha("blue", 0.5))
legend(2.5, 85, legend=c("Mammals", "Birds", "All Classes"),
       col=c("red", "blue", "gray"), lty=1:2, cex=0.8)
```

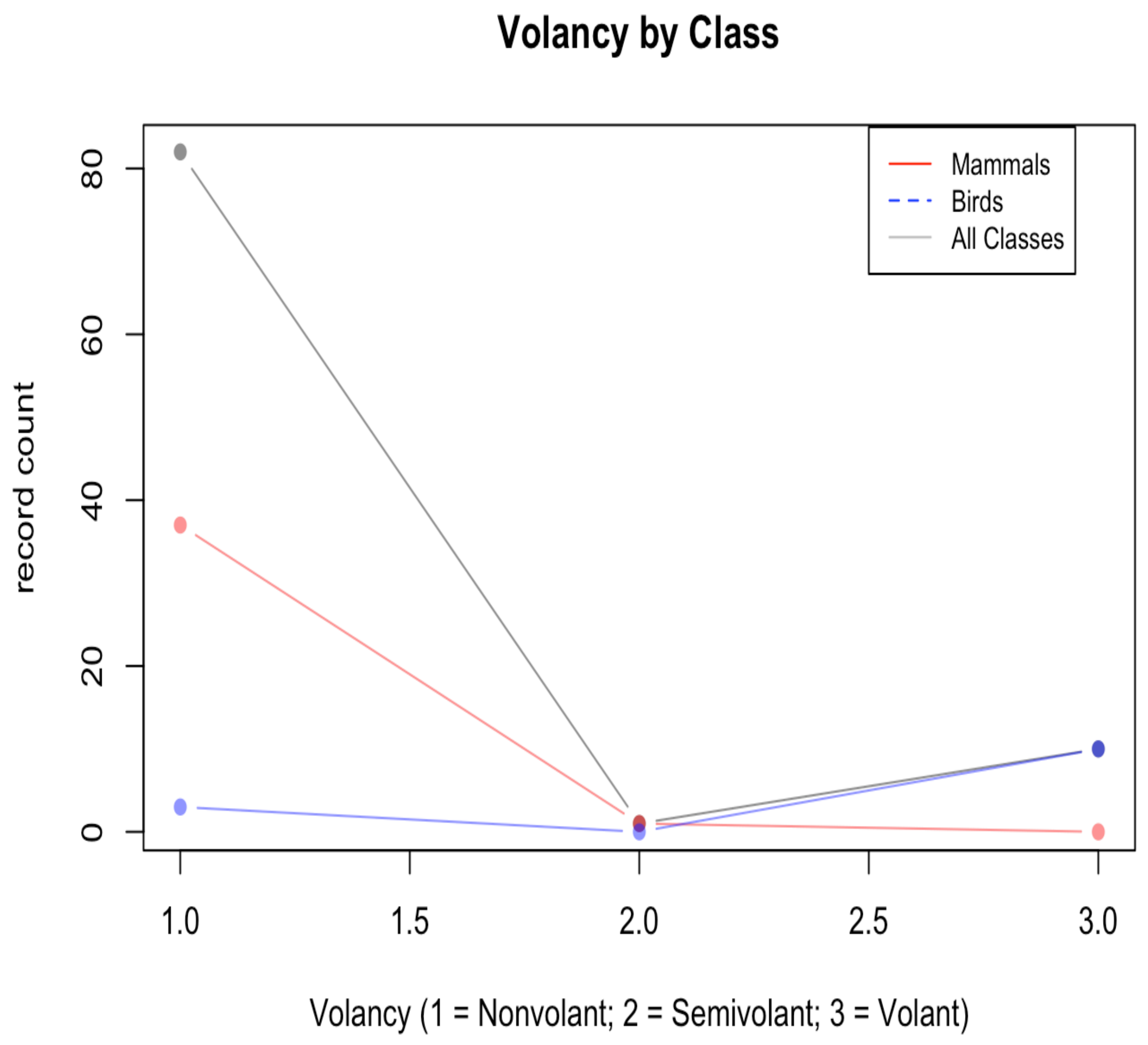

If one is more generally interested in the class breakdown of factors across a trait field without a priori interest in a specific variable, one can take advantage of the tools.

```
# Evaluating the variance breakdown within a given class


Volant_sum <- bio_vol %>%
  filter(volancy=="Volant")%>%
  group_by(Class, volancy)%>%
  count()


Semivolant_sum <- bio_vol %>%
```

```
  filter(volancy=="Semi-volant")%>%
  group_by(Class, volancy)%>%
  count()

Nonvolant_sum <- bio_vol %>%
  filter(volancy=="Non-volant")%>%
  group_by(Class, volancy)%>%
  count()

volTable <- Nonvolant_sum # Generating DF

volTable <- volTable[,-2]
colnames(volTable)[2] <- "Nonvolant"
volTable[,3] <- rep(0, length(Nonvolant_sum$Class))
volTable[,4] <- rep(0, length(Nonvolant_sum$Class))
colnames(volTable)[3] <- "Volant"
colnames(volTable)[4] <- "Semivolant"
volTable[match(Volant_sum$Class, Nonvolant_sum$Class),3] <- Volant_sum$n
volTable[match(Semivolant_sum$Class, Nonvolant_sum$Class),4] <- Semivolant_su
m$n

volTable
## # A tibble: 13 × 4
## # Groups:   Class [13]
##    Class          Nonvolant Volant Semivolant
##    <chr>              <int>  <dbl>      <dbl>
##  1 Actinopterygii         9      0          0
##  2 Amphibia               1      0          0
##  3 Anthozoa               5      0          0
##  4 Aves                   3     10          0
##  5 Bivalvia               3      0          0
##  6 Branchiopoda           2      0          0
##  7 Demospongiae           1      0          0
##  8 Elasmobranchii         1      0          0
##  9 Gastropoda             3      0          0
```

```
## 10 Insecta                 2       0           0
## 11 Mammalia               37       0           1
## 12 Reptilia                8       0           0
## 13 <NA>                    7       0           0
```

1241

Basic Regression using MOSAIC

http://mosaicdatabase.web.ox.ac.uk

Updated 22 February 2022

Vignette #3 - Basic Regression Using MOSAIC & COMPADRE

MOSAIC was built with the intention of its use in comparative biodemography - to strengthen the map of which functional traits predict vital rates and which do not. Traits in the MOSAIC database (factorial and numeric variables) are formatted to be easily accessed and used for regression-based analyses, among other types of analysis. MOSAIC was also designed for quick integration with the COMADRE, COMPADRE, and PADRINO structural population model databases, as outlined in the model exercise below.

```
library(devtools)
library(tidyverse)
library(Rcompadre) # package to access matrix population models
library(Rage) # package to perform life-history calculations
```

Accessing MOSAIC

Download MOSAIC from the mosaic portal. For more information on the basics of downloading MOSAIC and navigating the data structure, see: Vignette #1:

```
library(devtools) # Compulsory package to pull down packages from GitHub. Install if necessary.
install_github("mosaicdatabase/Rmosaic") # library of navigation-aiding functions
source_url("https://raw.githubusercontent.com/mosaicdatabase/mosaicdatabase/main/mosaic_fetch.R") # Link to GitHub repo
mosaic <- mos_fetch("v1.0.0") # Download version 1.0.0 (active version Feb 2022)
library(Rmosaic)
```

Regressing Biomass on Generation Time

In this exercise, the simple bivariate relationship of biomass on generation time is explored through basic regression. Bare in mind, that the below offer the building blocks for more complex multiple regression exercises and other forms of analysis - such as geospatial interpolation and ordination-based practices (see Vignette #4 for a brief vignette of PVA using mosaic).

#1: Extract biomass data for mammals

Extract the biomass values for mammals in the MOSAIC dataset.

```
# Extracting all biomass data

mosaic_dataframe <- data.frame(mosaic@taxaMetadat,
                               Biomass = as.numeric(mosaic@biomass@value),
                               MOSAIC_index = 1:length(mosaic@index))

# Restrict mammal data with non NA biomass data

mosaic_mammal <- subset(mosaic_dataframe,
                        Class == "Mammalia" & is.na(Biomass) == F)

# Extracting the first COMPADRE matrix id for each entry

matrix_ids_full <- mosaic@index[mosaic_mammal$MOSAIC_index]

# Extract just the first matrix id using sapply

mammal_matrix_ids <- sapply(matrix_ids_full, `[[`, 1)

# Add matrix id to the mammal data

mosaic_mammal$MatrixID <- mammal_matrix_ids

# Add full species names

mosaic_mammal$Binomial <- paste0(mosaic_mammal$Genus, " ", mosaic_mammal$Species)
```

#2: Download COMADRE matrix population models

A cdb_fetch() function is used to clone the Comadre database <**Error! Hyperlink reference not valid.** - which is an s3 copy of a SQL-based relational database - into an S4 data object locally manipulable in R.

Using ids associated with each matrix population model, we will subset the Comadre database of matrix population models for the animals. First we use the Rcompadre package to 'fetch' the Comadre database.

```
# Download the most recent database

comadre <- cdb_fetch("comadre")

# Rcompadre function flags potential issues with matrices, including NAs and ergodicity

comadre_flag <- cdb_flag(comadre)

# Remove matrices with NAs and those that are non-ergodic (which throw errors in generation time)

comadre_correct <- subset(comadre_flag,
                          check_NA_A == FALSE &
                          check_ergodic == TRUE)
```

The subsets of mosaic and Compadre can be overlapped in one line of code.

```
# Subset to mammal matrices

comadre_biomass <- subset(comadre_correct,
                          MatrixID %in% mammal_matrix_ids)
```

3. Calculating generation time

Rcompadre enables sub-setting of a matrix population model (life table) into constituent matrices representing the survival and fecundity/reproductive components (U and F matrices, respectively). Functions in the Rage package often require the specification of both the U and the F matrix, not the combined A matrix. The Rage package contains a suite of common demographic calculations - including GENERATION TIME - which are used to simplify this exercise. Note that other common demographic derivations, including transient indices can be extracted using the identical procedure.

```
# Calculate Generation Time

comadre_biomass$Generation_Time <- mapply(Rage::gen_time,
                                          matU(comadre_biomass),
```

```
                                                       matF(comadre_biomass))
```

4. Explore the relationship of generation time data and biomass data

Matrix IDs can be used to bridge generation time from comaadre with mosaic records.

```
# Extract matrix ID and generation time columns (excluding other matrix metadata)

comadre_biomass <- as.data.frame(comadre_biomass)

comadre_biomass <- comadre_biomass[,c("MatrixID", "Generation_Time")]

# Join the datasets, using Matrix IDs to crosswalk the data

mosaic_biomass <- merge(mosaic_mammal, comadre_biomass,
                        by = "MatrixID", all.x = TRUE)
# Remove infinity values or NA

mosaic_biomass <- mosaic_biomass[!(is.infinite(mosaic_biomass$Generation_Time) |
                                     is.na(mosaic_biomass$Generation_Time)),]

# Log biomass and generation time

mosaic_biomass$log10_biomass <- log10(mosaic_biomass$Biomass)
mosaic_biomass$log10_gent <- log10(mosaic_biomass$Generation_Time)



# The plot

plot(mosaic_biomass$log10_biomass, mosaic_biomass$log10_gent,
     pch=16, cex=1.5, col=alpha("black", 0.5),
     xlab=expression(paste(log[10], "Biomass")),
     ylab=expression(paste(log[10], "Generation time"))
```

```
)


# Simple linear regression (note that we are skipping model fitting in this b
rief vignette)

lmBioGenT = lm(log10_gent~log10_biomass, data = mosaic_biomass)

summary(lmBioGenT)
##
## Call:
## lm(formula = log10_gent ~ log10_biomass, data = mosaic_biomass)
##
## Residuals:
##      Min       1Q   Median       3Q      Max
## -0.53325 -0.16107 -0.01597  0.19936  0.38382
##
## Coefficients:
##               Estimate Std. Error t value Pr(>|t|)
## (Intercept)    0.85966    0.25372   3.388  0.00253 **
## log10_biomass  0.02762    0.05728   0.482  0.63418
## ---
## Signif. codes:  0 '***' 0.001 '**' 0.01 '*' 0.05 '.' 0.1 ' ' 1
##
## Residual standard error: 0.2623 on 23 degrees of freedom
## Multiple R-squared:  0.01001,    Adjusted R-squared:  -0.03303
## F-statistic: 0.2326 on 1 and 23 DF,  p-value: 0.6342
abline(lmBioGenT, col="red", lty=2, lwd=2)
```

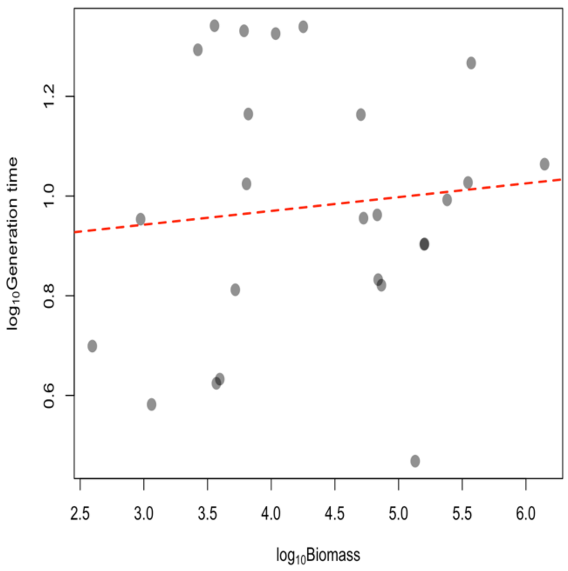
log$_{10}$Generation time
0.6
0.8
1.0
1.2
2.5
3.0
3.5
4.0
4.5
5.0
5.5
6.0
log$_{10}$Biomass

Dimension Reduction + MOSAIC

http://mosaicdatabase.web.ox.ac.uk

Updated 14 March 2022

Vignette #4 – Dimension Reduction + MOSAIC

MOSAIC was built for integrated analyses across trait fields. Whether the data are continuous (e.g., biomass) or categorical (e.g., dispersal class), MOSAIC allows researchers to explore these dimensions to identify the primary axes of variation across functional traits. Here, we will use a Multiple Factor Analysis (MFA) to explore the primary axes of variation between: biomass, dispersal capacity, growth determination, habitat type and mating system.

Note: These traits are picked solely to illustrate the analysis method that can be scaled for other traits in MOSAIC, demographic rates in COMADRE, COMPADRE and PADRINO and climate data from ERA5-Land.

```
library(devtools)
library(tidyverse)
library(FactoMineR) # Required for MFA.
library(factoextra) # Required for visualizing MFA.
```

Accessing MOSAIC

Download MOSAIC from the mosaic portal. For more information on the basics of downloading MOSAIC and navigating the data structure, see: Vignette #1:

```
remotes::install_github("mosaicdatabase/Rmosaic")
library(Rmosaic2.0)
mosaic <- mos_fetch("v1.0.0")
```

```
##
## Phylogenetic tree with 1359 tips and 1358 internal nodes.
##
## Tip labels:
##   Oxalis_acetosella, Rourea_induta, Euphorbia_telephioides, Euphorbia_fontqueriana, Triadica_sebifera, Actinostemon_concolor, ...
## Node labels:
##   Node1, Node2, Node3, Node4, Node5, Node6, ...
##
```

```
## Rooted; includes branch lengths.
```

Generating Dataframe

```
# Create a dataframe storing the values of the traits of interest.

mosaic_dataframe <- data.frame(mosaic@taxaMetadat,
                               Biomass = as.numeric(mosaic@biomass@value),
                               Growth_Determination = as.factor(mosaic@growth
det@value),
                               Mating_System = as.factor(mosaic@matsyst@value
),
                               Dispersal_Capacity = as.factor(mosaic@dispcap@
value),
                               Habitat_Type = as.factor(mosaic@aquadep@value)
)

# Create a dataframe that only contains data for MFA.
# This step makes all dimension reduction functions easier.

mosaic_dataframe_subset <-  mosaic_dataframe[,c(8:12)] %>%
  drop_na() %>%
  filter(Growth_Determination != "NDY") %>%
  filter(Mating_System != "NDY") %>%
  filter(Dispersal_Capacity != "NDY") %>%
  filter(Habitat_Type != "NDY")
```

Perform MFA

```
# Run the multiple factor analysis using the MFA() function from FactoMineR.

mfa_mosaic <- MFA(mosaic_dataframe_subset, group = c(1, 1, 1, 1, 1),
    type = c("s", rep("n", 4)),
    name.group=c("Biomass","Growth_Determination","Mating_System","Dispersal_
Capacity", "Habitat_Type"),
    graph = FALSE)

```

```
# Note: Changing the graph argument to TRUE will return some diagnostic graph
s of the MFA.
```

Explore MFA

In this hypothetical example, we are interested in what traits covary with the primary axes of variation in the MFA (i.e., Dim1 and Dim2). To visualize these relationships, we can use the in-built fviz functions in the factoextra package to illustrate (1) position of individuals, (2) average effect of trait level (i.e. monogamous) on individual position within the 2 dimensions and (3) average effect of trait type (i.e. mating system).

```
fviz_mfa_ind(mfa_mosaic,

             repel = TRUE,

             col.ind = "dark blue")
```

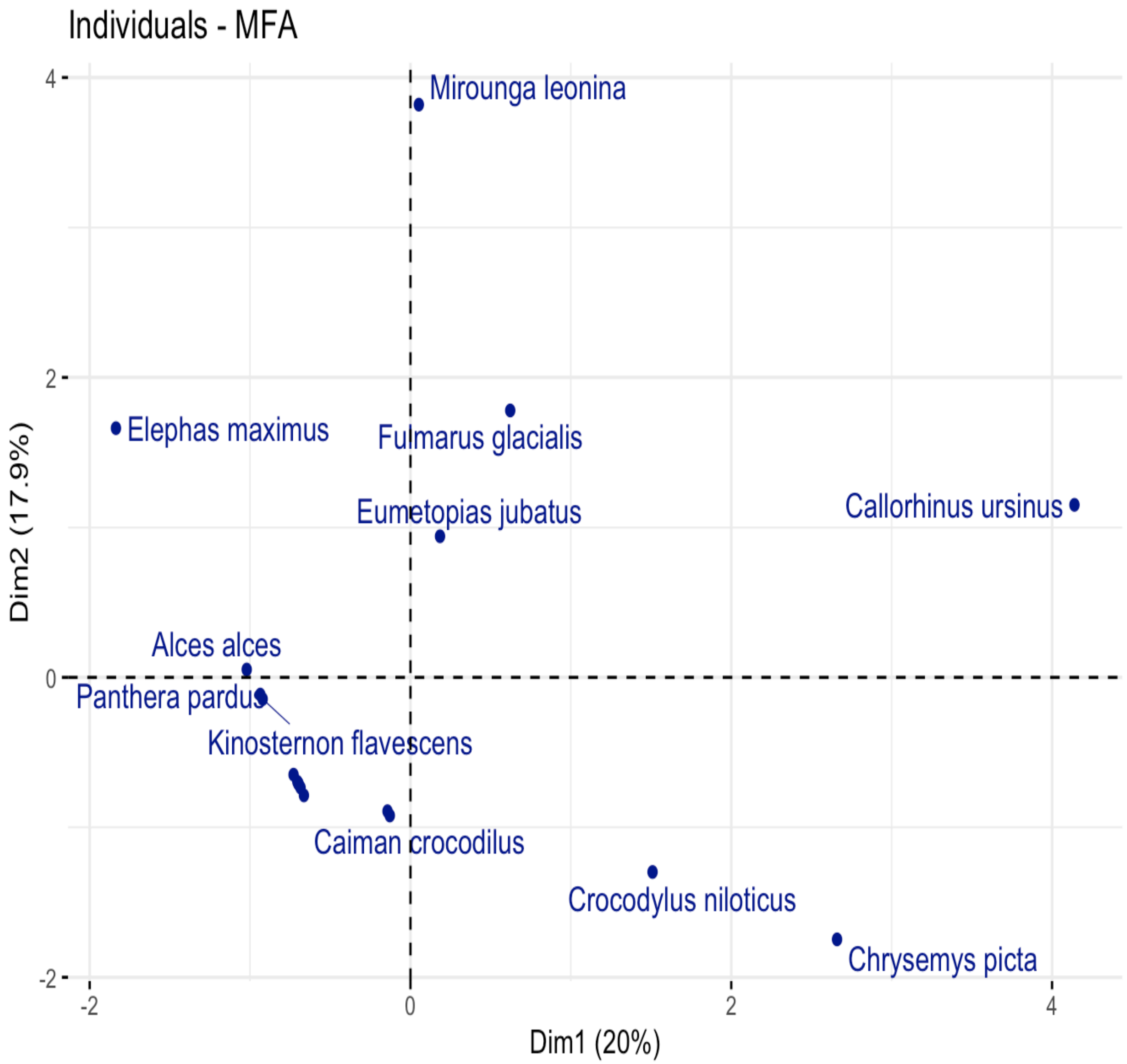


```
fviz_mfa_axes(mfa_mosaic,
              repel = TRUE)
```

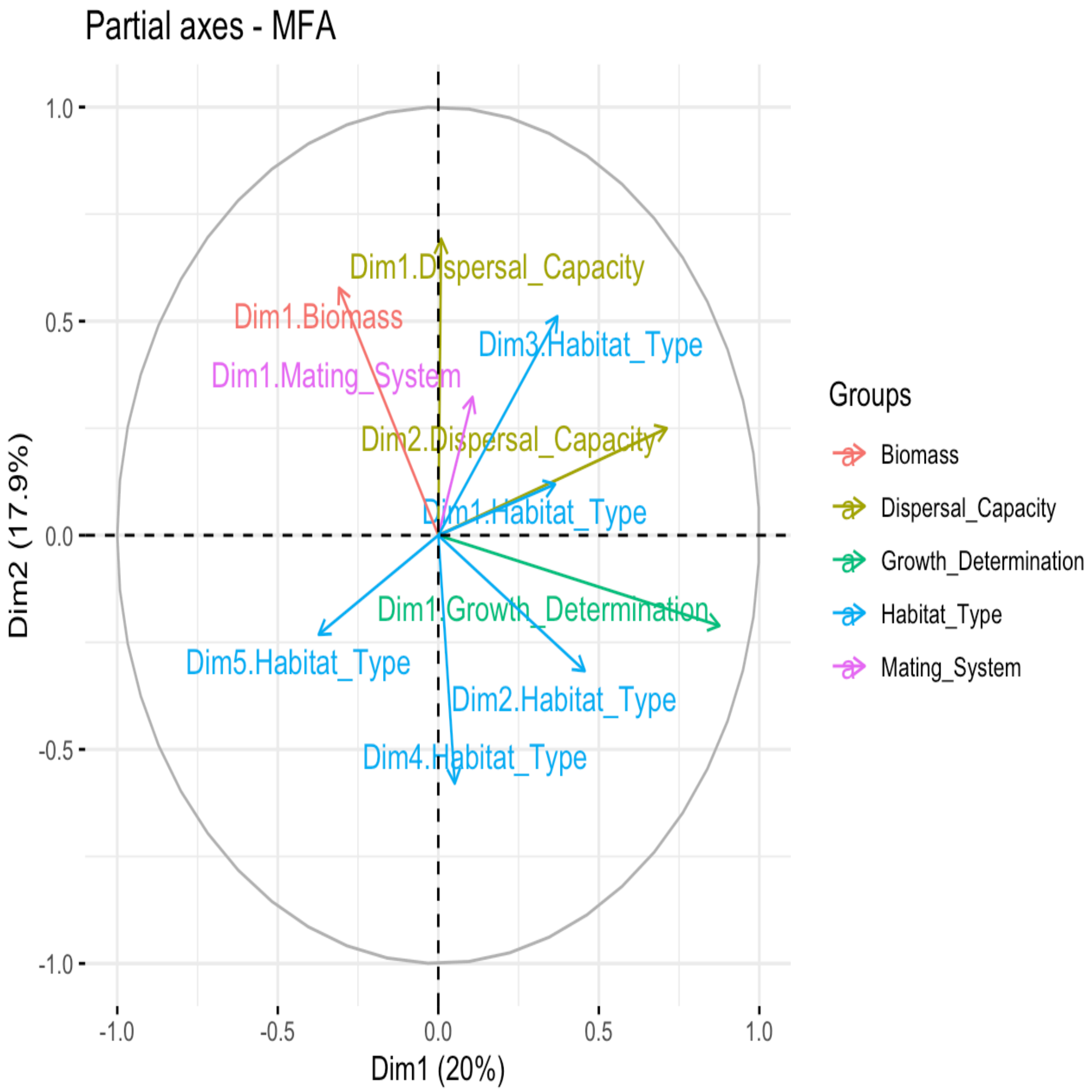


```
fviz_hmfa_var(mfa_mosaic,
              repel = TRUE,
              choice = "group",
              col.var = "dark blue")
```

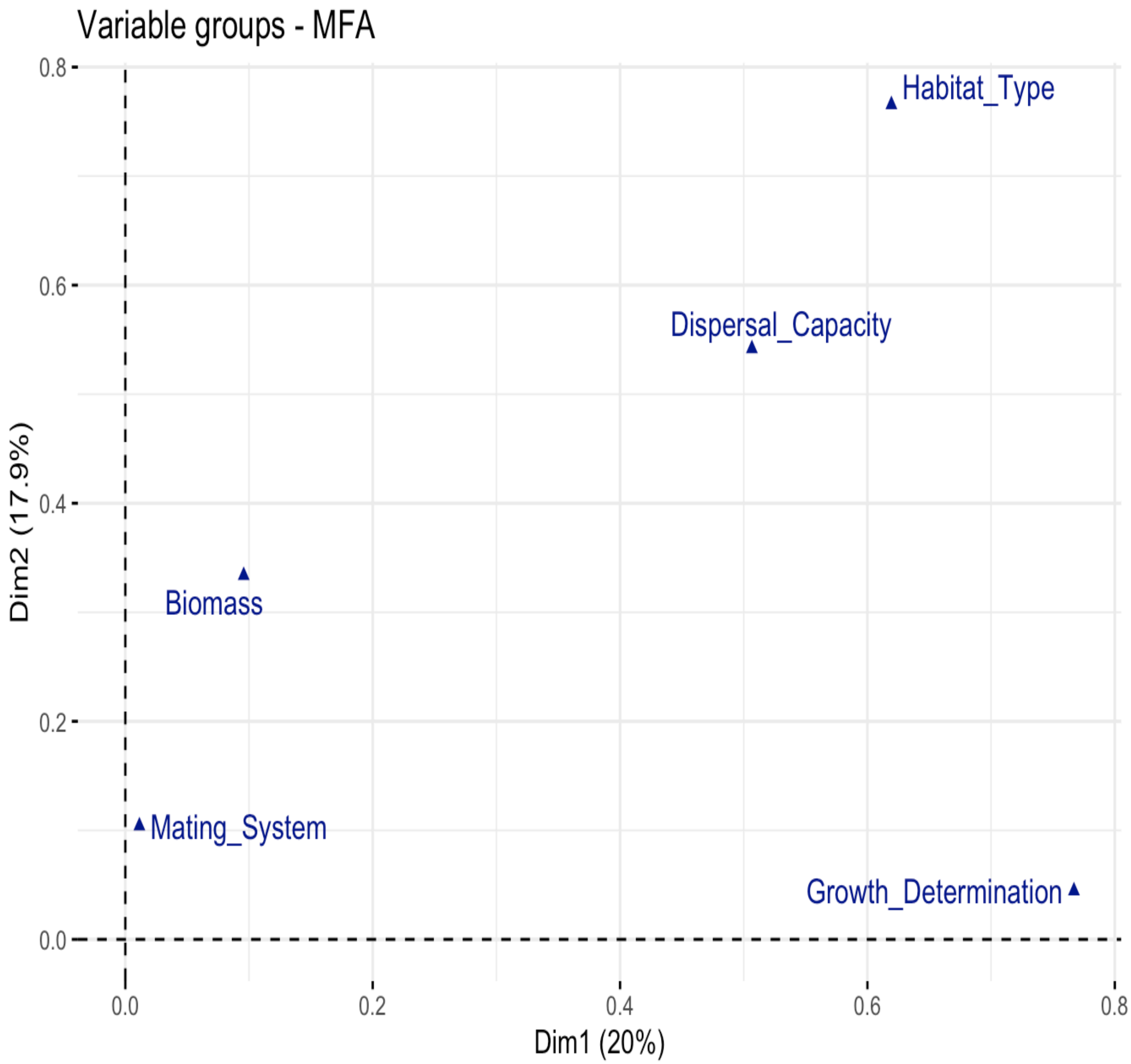
Variable groups - MFA
Habitat_Type
Dispersal_Capacity
Biomass
Mating_System
Growth_Determination
Dim2 (17.9%)
Dim1 (20%)
0.0
0.2
0.4
0.6
0.8

Climate Data + MOSAIC

http://mosaicdatabase.web.ox.ac.uk

Updated 14 March 2022

Vignette #5 – Climate Data + MOSAIC

MOSAIC was built with the intention of its use in comparative biodemography - to strengthen the map of which functional traits predict vital rates and which do not. Traits in the MOSAIC database (factorial and numeric variables) are formatted to be easily accessed and used for regression-based analyses, among other types of analysis. MOSAIC was also designed with the analysis of demographic metrics and traits in their environmental contexts. To this end, MOSAIC includes a separate object (climate) which we will access and make use of in the following exercise.

```
library(devtools)
library(tidyverse)
library(leaflet)
```

Accessing MOSAIC

Download MOSAIC from the mosaic portal. For more information on the basics of downloading MOSAIC and navigating the data structure, see: Vignette #1:

```
remotes::install_github("mosaicdatabase/Rmosaic")
library(Rmosaic2.0)
mosaic <- mos_fetch("v1.0.0")
```

```
##
## Phylogenetic tree with 1359 tips and 1358 internal nodes.
##
## Tip labels:
##   Oxalis_acetosella, Rourea_induta, Euphorbia_telephioides, Euphorbia_font
queriana, Triadica_sebifera, Actinostemon_concolor, ...
## Node labels:
##   Node1, Node2, Node3, Node4, Node5, Node6, ...
##
## Rooted; includes branch lengths.
```

Selecting Target Matrices

To get this exercise started, we first need to select which part of MOSAIC we are interested in. For simplicity, we are here focusing on mammal biomass as was done in Vignette #2:

```
# Extracting all biomass data
mosaic_dataframe <- data.frame(mosaic@taxaMetadat,
                               Biomass = as.numeric(mosaic@biomass@value),
                               MOSAIC_index = 1:length(mosaic@index))

# Restrict mammal data with non NA biomass data
mosaic_mammal <- subset(mosaic_dataframe,
                        Class == "Mammalia" & is.na(Biomass) == FALSE)

# Extracting the first COMPADRE matrix id for each entry
matrix_ids_full <- mosaic@index[mosaic_mammal$MOSAIC_index]

# Extract just the first matrix id using sapply
mammal_matrix_ids <- sapply(matrix_ids_full, `[[`, 1)

# Add matrix id to the mammal data
mosaic_mammal$MatrixID <- mammal_matrix_ids

# Add full species names
mosaic_mammal$Binomial <- paste0(mosaic_mammal$Genus, " ", mosaic_mammal$Species)
```

We now have a data frame containing MOSAIC IDs, taxonomic information, and biomass values for all mammals within MOSAIC. The corresponding matrix IDs are:

```
mammal_matrix_ids
##  [1] 249092 249094 240296 240307 249126 240331 249159 240358 240362 249214
## [11] 249248 240402 249269 249273 249275 249286 240470 249399 249409 249455
## [21] 249504 240502 249524 249526 249597 249598 240504 249671 249674 249723
## [31] 249726 240674 249753 249759 249787 240511 249817 249821 249866 240565
## [41] 249878 240677 249909 240645
```

Accessing the Climate Data

With our matrix IDs at hand for analysis, we are now ready to tap into the climate data pool contained within MOSAIC. The climate data hereafter, has been obtained using the R-package KrigR and climate data is reported as mean and standard deviation of monthly time-series belonging to the location and study duration for each matrix contained within MOSAIC.

Climate Data in MOSAIC

The climate data within MOSAIC is stored in the climate object:

```
head(mosaic@climate)
```

```
##   X.1 X MatrixID      Lat       Lon StudyStart StudyEnd     GenTime      d
b
## 1   1 1   240296 61.79944 -150.3694       1976     1986 10.64398229 comadr
e
## 2   2 2   240297 61.79944 -150.3694       1976     1986        <NA> comadr
e
## 3   3 3   240298 61.79944 -150.3694       1976     1986        <NA> comadr
e
## 4   4 4   240299 61.79944 -150.3694       1976     1986        <NA> comadr
e
## 5   5 5   240300 61.79944 -150.3694       1976     1986  8.16781721 comadr
e
## 6   6 6   240301 61.79944 -150.3694       1976     1986  12.2931104 comadr
e
##   Download air_temperature air_temperature_SD total_precipitation
## 1        1        273.7485           9.862699          0.06464312
## 2        1        273.7485           9.862699          0.06464312
## 3        1        273.7485           9.862699          0.06464312
## 4        1        273.7485           9.862699          0.06464312
## 5        1        273.7485           9.862699          0.06464312
## 6        1        273.7485           9.862699          0.06464312
##   total_precipitation_SD volumetric_soil_water_layer_1
## 1              0.0420547                     0.3704328
## 2              0.0420547                     0.3704328
## 3              0.0420547                     0.3704328
## 4              0.0420547                     0.3704328
## 5              0.0420547                     0.3704328
## 6              0.0420547                     0.3704328
##   volumetric_soil_water_layer_1_SD volumetric_soil_water_layer_2
## 1                       0.02808088                     0.3684833
```

```
## 2                   0.02808088                        0.3684833
## 3                   0.02808088                        0.3684833
## 4                   0.02808088                        0.3684833
## 5                   0.02808088                        0.3684833
## 6                   0.02808088                        0.3684833
##   volumetric_soil_water_layer_2_SD volumetric_soil_water_layer_3
## 1                   0.02796726                          0.36928
## 2                   0.02796726                          0.36928
## 3                   0.02796726                          0.36928
## 4                   0.02796726                          0.36928
## 5                   0.02796726                          0.36928
## 6                   0.02796726                          0.36928
##   volumetric_soil_water_layer_3_SD volumetric_soil_water_layer_4
## 1                   0.02442513                        0.3888525
## 2                   0.02442513                        0.3888525
## 3                   0.02442513                        0.3888525
## 4                   0.02442513                        0.3888525
## 5                   0.02442513                        0.3888525
## 6                   0.02442513                        0.3888525
##   volumetric_soil_water_layer_4_SD     runoff  runoff_SD total_evaporation
## 1                   0.01339378 0.03377308 0.02755258       -0.02956482
## 2                   0.01339378 0.03377308 0.02755258       -0.02956482
## 3                   0.01339378 0.03377308 0.02755258       -0.02956482
## 4                   0.01339378 0.03377308 0.02755258       -0.02956482
## 5                   0.01339378 0.03377308 0.02755258       -0.02956482
## 6                   0.01339378 0.03377308 0.02755258       -0.02956482
##   total_evaporation_SD
## 1           0.03250795
## 2           0.03250795
## 3           0.03250795
## 4           0.03250795
## 5           0.03250795
## 6           0.03250795
```

As you can see, there are NA values for some matrix IDs in the above output. This results from either (1) missing geolocations of the underlying matrix models making extraction of environmental parameters impossible, (2) study durations of matrix models pre-dating climate data availability at monthly scale within ERA5-Land, or (3) geolocation of matrix models falling outside of land masses.

Extracting Climate Data

Since we are only interested in a subset of matrix models, we do not require the full climate data object and so we can refer to only the relevant subset of the climate object by matching MatrixID values:

```
clim_df <- mosaic@climate[mosaic@climate$MatrixID %in% mammal_matrix_ids, ]
```

With the subsetted climate data, we can now create a data frame holding all necessary data for some exploratory analyses:

```
analysis_df <- merge(mosaic_mammal, clim_df, by = "MatrixID")
analysis_df <- na.omit(analysis_df[analysis_df$Biomass < 1e6, ]) # we set a cutoff on biomass here, to avoid influence of outliers. This is just a procedure for simplicity of this exercise
```

Let’s plot the remaining data out in their geospatial context:

```
cols <- rainbow(n = length(unique(analysis_df$Order)))
names(cols) <- unique(analysis_df$Order)
analysis_df$Col <- cols[match(analysis_df$Order, names(cols))]

Map <- leaflet(analysis_df, width = "100%")
Map <- addProviderTiles(Map, providers$Esri.WorldTopoMap)
Map <- addCircleMarkers(map = Map, lng = ~Lon, lat = ~Lat,
                        label = ~Order,
                        col = ~Col,
                        labelOptions = labelOptions(textsize = "12px")
)
Map
```

Regression & Analysis

Now we are ready to run analyses. The ones we are focussing on here are rudimentary at best and should simply serve as a demonstration of how easily one can make use of state-of-the-art ERA5-Land climate data with the MOSAIC data base.

All Mammals

First, let's assess the effect of temperature on biomass of all mammals:

```
summary(lm(data = analysis_df, Biomass ~ air_temperature))
##
## Call:
## lm(formula = Biomass ~ air_temperature, data = analysis_df)
##
## Residuals
##     Min      1Q  Median      3Q     Max
## -136457  -35516  -12225   17982  218728
##
## Coefficients:
##                 Estimate Std. Error t value Pr(>|t|)
## (Intercept)      1502659     532349   2.823  0.00991 **
## air_temperature    -5006       1850  -2.706  0.01290 *
## ---
## Signif. codes:  0 '***' 0.001 '**' 0.01 '*' 0.05 '.' 0.1 ' ' 1
##
## Residual standard error: 79270 on 22 degrees of freedom
## Multiple R-squared:  0.2498, Adjusted R-squared:  0.2157
## F-statistic: 7.324 on 1 and 22 DF,  p-value: 0.0129
```

According to this, mammals in colder regions are heavier. This is in support of Bergmann's rule.

```
ggplot(data = analysis_df, aes(x = air_temperature, y = Biomass)) +
  geom_point() +
  stat_smooth(method = "lm") +
  labs(x = "Air Temperature [K]", y = "Biomass") +
  theme_bw()
```

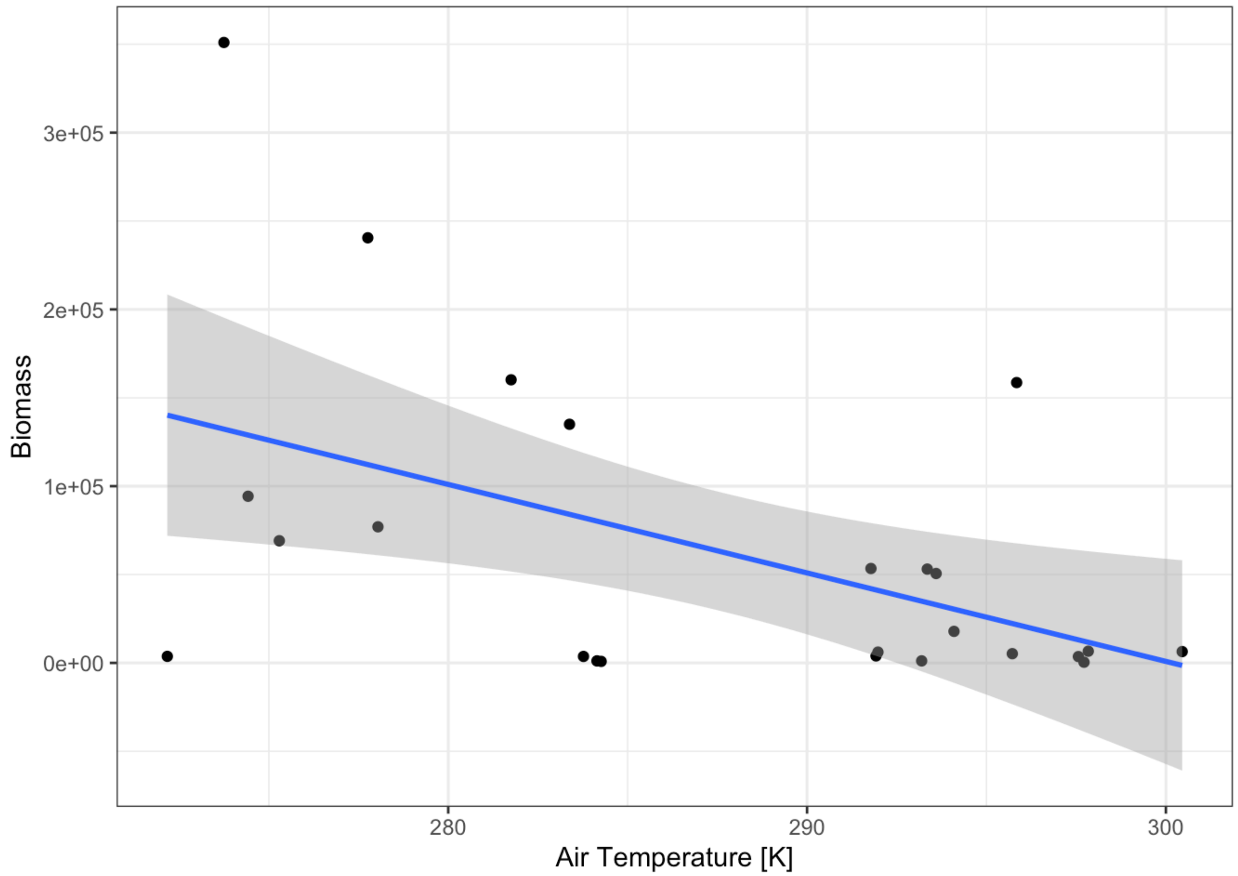

Mammals by Order

Now let’s make use of the higher-resolution taxonomic information available in MOSAIC and assess the impact of air temperature on biomass by order of mammals:

```
summary(lm(data = analysis_df, Biomass ~ air_temperature * Order))
```

```
##
## Call:
## lm(formula = Biomass ~ air_temperature * Order, data = analysis_df)
##
## Residuals:
##     Min      1Q  Median      3Q     Max
## -122023  -10392       0   12873  143838
##
## Coefficients: (4 not defined because of singularities)
##                                          Estimate Std. Error t value Pr(>|t|)
## (Intercept)                               3047458    2548979   1.196    0.255
```

```
## air_temperature                         -10376       9154  -1.133    0.279
## OrderCarnivora                        -1985731    2765125  -0.718    0.486
## OrderCingulata                          -14654     148341  -0.099    0.923
## OrderDidelphimorphia                     -4262     158154  -0.027    0.979
## OrderDiprotodontia                    -3150686    3435359  -0.917    0.377
## OrderLagomorpha                         -99518      95702  -1.040    0.319
## OrderPrimates                         -2008837    3738418  -0.537    0.601
## OrderRodentia                          -219816     100383  -2.190    0.049
*
## air_temperature:OrderCarnivora            7092       9885   0.717    0.487
## air_temperature:OrderCingulata              NA         NA      NA       NA
## air_temperature:OrderDidelphimorphia        NA         NA      NA       NA
## air_temperature:OrderDiprotodontia       10742      12153   0.884    0.394
## air_temperature:OrderLagomorpha             NA         NA      NA       NA
## air_temperature:OrderPrimates             6911      13007   0.531    0.605
## air_temperature:OrderRodentia               NA         NA      NA       NA
## ---
## Signif. codes:  0 '***' 0.001 '**' 0.01 '*' 0.05 '.' 0.1 ' ' 1
##
## Residual standard error: 75170 on 12 degrees of freedom
## Multiple R-squared:  0.6319, Adjusted R-squared:  0.2945
## F-statistic: 1.873 on 11 and 12 DF,  p-value: 0.148
```

Well, this is just a mess, but should serve to illustrate how many different analyses can easily be performed with MOSAIC and the in-built climate parameters.

```
ggplot(data = analysis_df, aes(x = air_temperature, y = Biomass, col = Order)
) +
  geom_point() +
  stat_smooth(method = "lm") +
  labs(x = "Air Temperature [K]", y = "Biomass") +
  theme_bw() + scale_color_manual(values = cols)
```

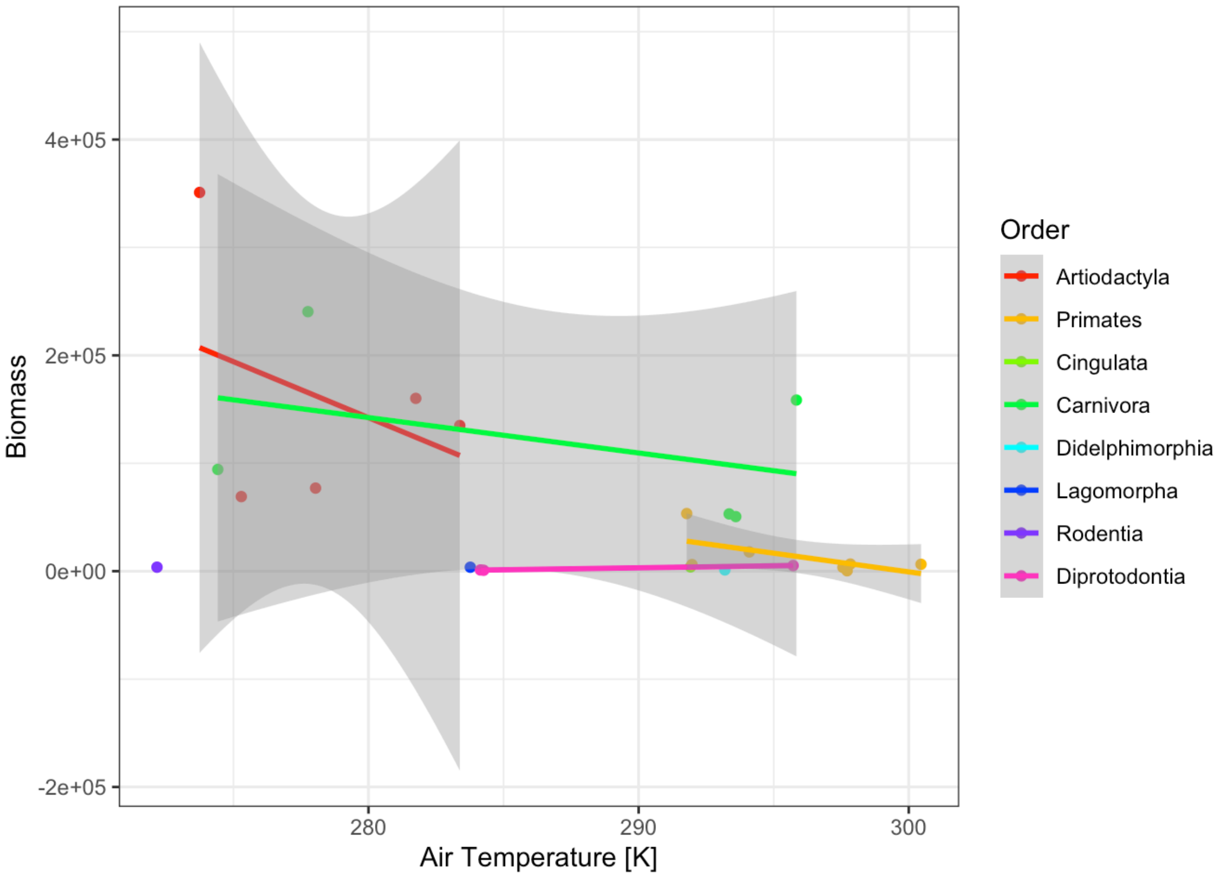
Biomass
4e+05
2e+05
0e+00
-2e+05
280
290
300
Air Temperature [K]
Order
Artiodactyla
Primates
Cingulata
Carnivora
Didelphimorphia
Lagomorpha
Rodentia
Diprotodontia

S6: LogNormal Distribution of Mass and Height

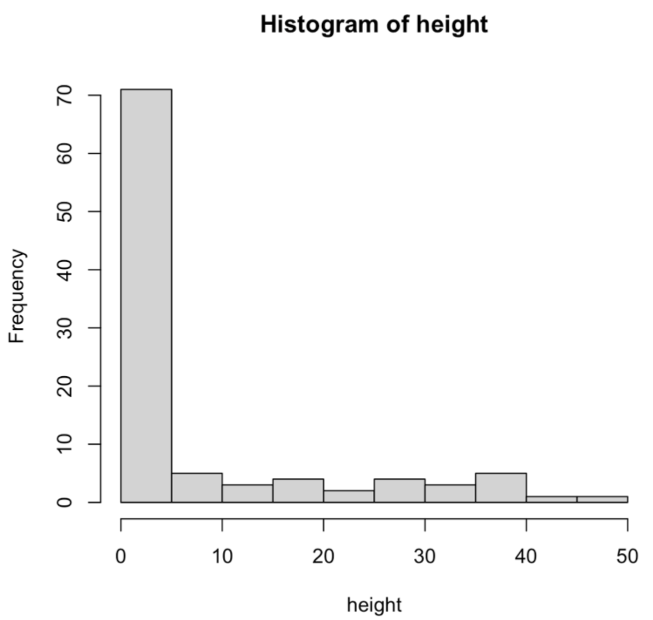

Raw heights from MOSAIC v1.0.0

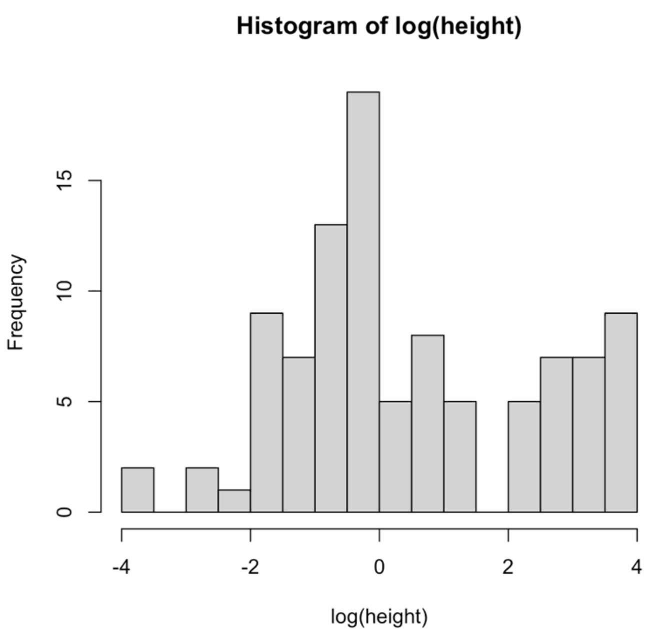

Log Heights from MOSAIC v.1.0.0

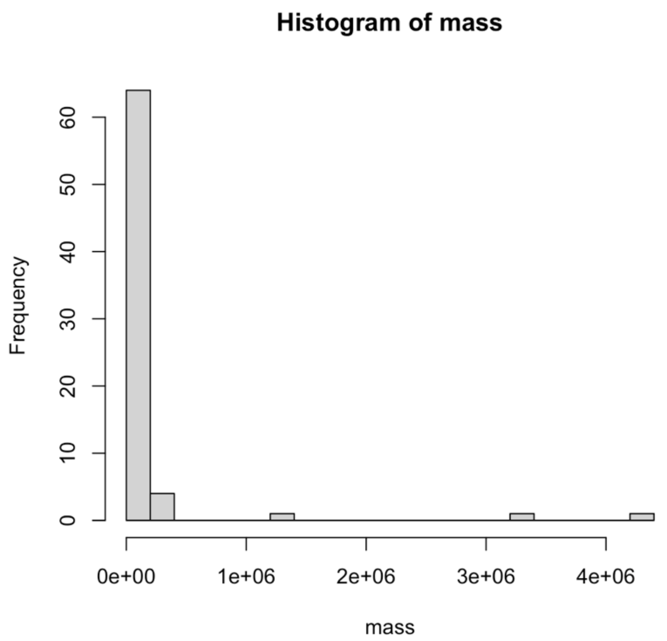

Raw mass from MOSAIV v1.0.0

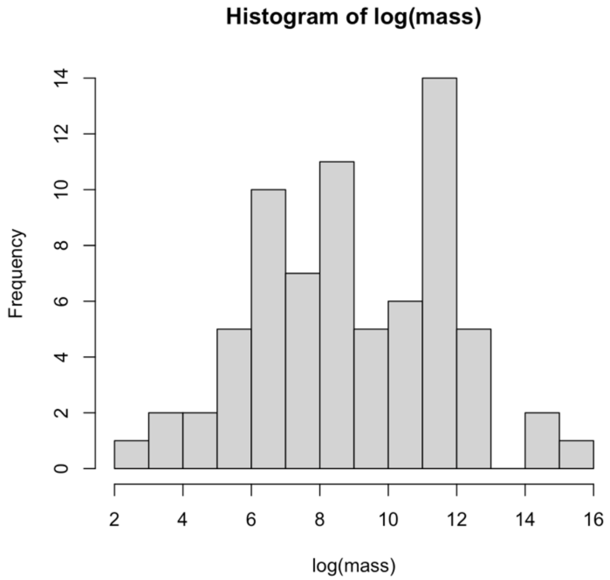

Log mass from MOSAIC v1.0.0
